# HIV Disclosure: HIV-positive status disclosure to sexual partners among individuals receiving HIV care in Addis Ababa, Ethiopia

**DOI:** 10.1371/journal.pone.0211967

**Published:** 2019-02-15

**Authors:** Noah G. Dessalegn, Rahel G. Hailemichael, Aster Shewa-amare, Shailendra Sawleshwarkar, Bereket Lodebo, Alemayehu Amberbir, Richard J. Hillman

**Affiliations:** 1 The University of Sydney Westmead Clinical School, Faculty of Medicine and Health, University of Sydney, Sydney, Australia; 2 Myungsung Medical College, Addis Ababa, Ethiopia; 3 Zewditu Memorial Hospital, Addis Ababa, Ethiopia; 4 Wellstar Atlanta Medical Centre, Atlanta, Georgia, United States of America; 5 Dignitas International, Medical and Research Department, Zomba, Malawi; 6 Western Sydney Sexual Health Centre, Western Sydney Local Health District, Sydney Medical School, Parramatta, New South Wales, Australia; 7 Centre for Applied Medical Research, St Vincent's Hospital, Darlinghurst, Sydney, New South Wales, Australia; Pontificia Universidade Catolica do Rio Grande do Sul, BRAZIL

## Abstract

**Introduction:**

Disclosure of HIV status to sexual partners can help HIV prevention efforts and enable HIV positive people to receive social support, as well as increasing access and adherence to treatment. This study was conducted to determine the rate, processes, outcomes, and correlates of HIV status disclosure to sexual partners among HIV positive individuals.

**Method:**

A cross-sectional study was conducted between September and November 2015 at two HIV outpatient clinics in Addis Ababa, Ethiopia. Data were collected using an interviewer- administered semi-structured questionnaire. Logistic analysis was used to determine the independent correlates of serostatus disclosure.

**Results:**

Of 742 participants, (371 men and 371 women), 727 (98%) were on antiretroviral therapy and 676 (91.1%) had at least one sexual partner since their HIV diagnosis, of whom 558 (82.5%) had disclosed their HIV status to their most recent sexual partner. Of those who reported having disclosed their status to their most recent sexual partner, 82 (14.7%) had at least one unprotected sexual encounter with the partner, after HIV diagnosis but prior to disclosure. The most commonly reported initial outcome of disclosure was gaining emotional and/or financial support. Some (11.3%) also reported that their disclosure immediately initiated their partner for HIV testing. Negative outcomes to disclosure, such as stigma and discrimination, were more common among females (26.2%) compared to males (12.7%). In the multiple regression analysis, disclosure was associated with greater condom use, greater social support, knowing the partner’s HIV status, having a good relationship with the partner, and cohabiting with the partner.

**Conclusion:**

HIV disclosure was common amongst participants, although sometimes delayed, with possible consequences for onward transmission. Fear of negative outcomes, such as verbal abuse and physical violence, were major barriers to disclosure. Efforts to support disclosure have the potential to contribute to HIV control and prevention by encouraging safer sexual practice, initiating partners for HIV testing, and enhancing support for people living with HIV.

## Introduction

HIV/AIDS remains one of the world’s serious public health challenges [1]. Eastern and Southern Africa accounts for 45% of new HIV infections in the world and is home to 53% of people living with HIV globally in 2017; which makes it the region most affected by the HIV epidemic. In 2017, 2% of all global new HIV infections and 3% of global HIV related deaths occurred in Ethiopia [2]. The national prevalence in adults aged 15–49 years in 2017 was 0.9% (0.7% in males and 1.2% in females) [3].

In the context of HIV prevention, “disclosure” is defined as the process of revealing HIV positive status to sexual partner(s), family members, or others in their social circle and typically occurs gradually, over time[4]. Disclosure has the potential to play significant roles in both HIV prevention (through reducing onward transmission) and management (by facilitating greater social support and increased adherence). This is particularly relevant to countries such as Ethiopia, where the majority of HIV infections occurs through sexual transmission[5] and 72.7% of HIV affected couples (one or both infected) are serodiscordant [6].

The rate of HIV disclosure varies with location, as well as the subgroup of people living with HIV (PLWH) and the potential confidante. For example, a recent review of women’s disclosure to sexual partners[7] demonstrated differences between the developed world (average 71%; range: 42%-100%) and the developing world (average 52%; range: 16%-86%). A 2005 study from semi-rural South-West Ethiopia showed that 69% of HIV positive women had disclosed to their sexual partner[8]. A more recent study of HIV positive pregnant women[9] in Addis Ababa found that 73% of women had disclosed to their sexual partners, 65.4% within four weeks of their HIV diagnosis.

Compared to non-disclosure, disclosure by PLWH has been found to be associated with less anxiety, fewer symptoms of depression, and increased social support for the disclosing person. Most notably, disclosure was associated with strengthening of relationships [7, 10]. However, disclosure also entails potential risks. For example, it can result in loss of economic support, blame, abandonment, physical and emotional abuse, discrimination, and disruption of family relationships [7, 11].

A potential dilemma exists between respecting the privacy and human rights of PLWH, versus the need to protect at risk partners [4, 12]. Countries around the world have different HIV disclosure laws. In Ethiopia; HIV non-disclosure, exposure, and transmission criminalization laws fall under the 2004 revision of the criminal code of Ethiopia (Article 514 of the Penal Code / Spreading of Human Diseases). This Article prohibits intentional transmission of a communicable human disease (actual transmission, rather than exposure is considered the crime) [13, 14]. Additionally, the “Guideline for HIV Counselling and Testing in Ethiopia” also requires the health professional to disclose HIV status of a client if they judge that the partner is at risk by stating, “if a client fails to disclose after repeated documented counselling sessions (2–3 within two weeks) and the counsellor feels the partner is at risk of infection, the counsellor should consult the supervisor, senior or immediate management staff for further action including revealing the result” [15].

Addis Ababa has the second highest HIV prevalence among all Ethiopian regions [6]. In order to identify those most at risk of non-disclosure, and to determine optimum strategies to facilitate disclosure, we investigated the rate, processes, outcomes and factors influencing disclosure amongst attendees at these hospitals.

## Methods

### Study design and setting

The study was conducted between September and November 2015 in two public HIV/AIDS outpatient treatment clinics in Zewditu Memorial Hospital and Yekatit 12 Hospital in Addis Ababa, Ethiopia. In accordance with the national guideline, the two clinics provide testing and treatment of sexually transmitted infections, comprehensive HIV Counselling and Testing, Prevention of Mother to Child Transmission of HIV, and Antiretroviral treatment. Zewditu Memorial Hospital started the country’s first Antiretroviral treatment program in 2003. On record review as of September 2015, 7934 (3546 males and 4388 females) and 4012 (1459 males and 2553 females) HIV positive individuals 18 years and up are on ongoing HIV care and support in Zewditu Memorial Hospital and Yekatit 12 hospitals respectively. These two hospitals care for the largest number of HIV patients in Ethiopia and their disclosure characteristics have never been previously investigated. All participants were PLWH aged 18 or over, who had been attending one of the HIV clinics for at least two weeks prior to participation.

### Sample size and sampling frame

Prevalence of disclosure among women in Addis Ababa had previously been found to be 63.8% [16]. Thus, to detect at least a 10% difference in the rate of disclosure between men and women at 95% confidence level, 80% power, and adding 10% for non-response, the sample size was determined to be 337 in each gender group, resulting in a total sample size of 742. Using the appointment register of the hospitals as sampling frame, systematic random sampling was used to recruit the required number of participants into the study.

### Measurements

Participants were approached as they come for their regular consultation. For illiterate participants, the “Participant’s Information Statement” and “Participant Consent Form” were read out by the data collectors. Written informed consent was obtained from all participants, which was documented using signatures for literate participants and thumb impressions for illiterate ones. Data were collected using pre-tested semi-structured questionnaires, adapted from previous, similar studies **[17–19].** The questionnaire included sociodemographic and health status information, partnership status, healthcare service usage, sexual behaviour and psychosocial variables. Dependent and independent variables were measured as follows:

Disclosure was identified using the question “does your most recent sexual partner know your HIV status?”, and answers recorded as “yes” or “no”. Therefore, in this paper, “partner” refers to the most recent sexual partner participants had and “disclosure” refers to disclosure to this most recent sexual partner. The questionnaire did not ask the gender of participants’ sexual partners. If a participant indicated that s/he did not disclose to their most recent sexual partner, the reasons for non-disclosure were investigated. If an individual indicated that s/he had disclosed their status to their partner; reasons, process (methods), and outcomes (reactions) of the disclosure were investigated. To see the timeliness of the disclosure, the respondents were also asked the following question, “After you found out that you were infected, did you have unprotected sex with your partner before telling him/her about your infection?”, and answer was recorded as ‘yes’ or “no”. Participants who responded ‘yes’ were classified as having delayed disclosure. However, the questionnaire did not ask how long they stayed without disclosing.

The wealth index used was adapted from the 2011 version of the Demographic and Health Survey in Ethiopia [20] and a previous Ethiopian study [21]. Respondents were asked whether or not they owned each of a total of 12 different socioeconomic items (household items) and their answers were recorded as “Yes” or “No”. A score, ranging from 1 to 12, was obtained for each respondent, and data distribution was classified into three categories–poor, middle-class, and rich, using principal component analysis.

Self-reported adherence: based on practicality and methods used in various other studies [22–24], this was measured by identifying whether any dose(s) of antiretrovirals had been missed or not taken at the right time in the previous week. A person claiming to have taken 95% or more of the medicines within 2 hours of the correct time in the previous week was defined as having optimum adherence [25, 26]. Social support was measured using the Oslo Social Support 3-item scale (OSS-3), where a higher number reflects greater social support [27]. Depression was measured using the Patient Health Questionnaire 9 (PHQ 9) [28].

To measure quality of relationship prior to disclosure, participants were asked, “How do you see the relation with your most recent partner (until disclosure, *if participant indicated that s/he had disclosed*)?” It was a multi-response question, and the result was recorded as “peaceful”, “We quarrelled only sometimes”, “We often quarrelled”, or “We often quarrelled and have (are going to) separate(d)”. Participants who answered “peaceful” or “We quarrelled only sometimes” were defined to have “mostly peaceful relationship”. Participants who answered, “We often quarrelled” or “We often quarrelled and have (are going to) separate(d)” were defined to have “mostly quarrelsome relationship. We left this out to keep the manuscript concise but is now added under “Measurements” section.

Perceive HIV related stigma: stigma was assessed by asking subjects what they felt and how they are treated with varying levels of social contact. Over all 23 questions were asked to measure stigma which was sub-divided in to 10 questions about negative self-image, 10 questions about negative public attitude, and 7 questions about disclosure related stress. A summary indicator was calculated to dichotomize measure of stigma. Individuals who scored above the mode value were considered to have “high level of perceived stigma”.

### Data analysis

First, descriptive analysis was performed. Then bivariate analysis was carried out and explanatory variables found to be associated with the outcome variable in bivariate analyses were included in a multivariate logistic model to appreciate the adjusted effect and derive adjusted odds ratio of each of the dichotomous independent variables. The models were evaluated using backwards stepwise logistic regression technique. A 95% level of confidence was applied, and p-value was reported. Analysis was performed using SPSS version 22 for windows.

### Ethics approval

Ethical approval was obtained from Addis Ababa Health Bureau Ethics Review Committee and Sydney University Human Research Ethics Committee (Project No: 2015:200).

## Results

### Socio-demographic and partnership characteristics

Of 742 participants, (371 men and 371 women) approached, all (100%) consented and 727 (98%) were on ART. Table 1 summarizes the socio-demographic characteristics of participants. The median age was 39 years (range 21 to 76) and the majority, 619 (83.7%), were between 21 and 49 years. Overall, 652 (88.8%) were literate, 551 (75.5%) were employed, and 359 (48.4%) were in the lowest two quintiles of the Wealth Index (“poor category”).

**Table 1:** Socio-demographic characteristics of participants by gender.

Variable	Total (n = 742)	Male (n = 371)	Female (n = 371)
	N (%)	N (%)	N (%)
**Age (Years)**			
20–29	55 (7.5)	15 (4.1)	40 (10.9)
30–39	319 (43.0)	112 (30.4)	207 (56.3)
40–49	245 (33.2)	155 (42.0)	90 (24.5)
50–59	92 (12.5)	66 (17.9)	26 (7.1)
> 60	26 (3.5)	21 (1.4)	5 (1.5)
**Highest level of Education**			
No education	82 (11.2)	21 (5.7)	61 (16.5)
Primary	187 (25.5)	84 (23.0)	103 (27.9)
Secondary	280 (38.1)	140 (38.4)	140 (37.9)
Certificate and diploma	125 (17.0)	78 (21.4)	47 (12.7)
Degree and above	60 (8.2)	42 (11.5)	18 (4.9)
**Religion**			
Coptic Orthodox	561 (76.3)	289 (77.9)	272 (74.7)
Protestant	115 (15.6)	49 (13.2)	66 (18.1)
Muslim	49 (6.7)	29 (7.8)	20 (5.5)
Others	10 (1.4)	4 (1.1)	6 (1.6)
**Ethnicity**			
Amhara	358 (49.2)	174 (47.9)	184 (50.5)
Oromo	161 (22.1)	76 (20.9)	85 (23.4)
Tigre	129 (17.7)	71 (19.6)	58 (15.9)
Guraghe	47 (6.5)	24 (6.6)	23 (6.3)
Others	32 (4.4)	18 (5.0)	14 (3.8)
**Employment**			
Employed	551 (75.5)	314 (85.8)	237 (65.1)
**Wealth Index**			
Rich (highest 2 quintiles)	278 (37.5)	145 (39.1)	133 (35.8)
Middle class (3^rd^ quintile)	105 (14.2)	47 (12.7)	58 (15.6)
Poor (lowest 2 quintiles)	359 (48.4)	179 (48.2)	180 (48.5)

### Partnership, medical, behavioural, and psychosocial characteristics

As can be seen in Table 2, a large majority, 676 (91.1%), of participants had a sexual partner since becoming aware of their HIV positive status; of whom 151 (22.4%) reported being aware that they were in serodiscordant relationships at the time of the study and only 268 (46.0%) indicated that they always used condoms.

**Table 2:** Partnership, medical, and psycho-social characteristics of participants, by gender.

Variables	Total	Male	Female
	N (%)	N (%)	N (%)
**Had sexual partner after knowing HIV status (n = 742)**			
Yes	676 (91.1)	340 (91.6)	336 (90.6)
**Partnership status** ^μ^ **(n = 669)**			
Spouse	575 (85.9)	283 (84.7)	292 (87.2)
Steady sexual partner	57 (8.5)	23 (6.9)	34 (10.1)
Casual sexual partner	37 (5.5)	28 (8.4)	9 (2.7)
Not answered + missing data	7	6	1
**Co-habitation status** ^μ^ **(n = 670)**			
Living together	574 (85.7)	281 (83.9)	293 (87.5)
Not living together	96 (14.3)	54 (16.1)	42 (12.5)
Not answered + missing data	6	5	1
**Quality of relationship** ^μ^ **(n = 676)**			
Mostly peaceful	495 (73.2)	274 (80.6)	221 (65.8)
Mostly quarrelsome	181 (26.8)	66 (19.4)	115 (34.2)
**Partner HIV status** ^μ^ **(n = 675)**			
Positive	383 (56.7)	186 (54.9)	197 (58.6)
Negative	151 (22.4)	86 (25.4)	65 (19.3)
Unknown	141 (20.9)	67 (19.8)	74 (22.0)
**Type of HTC**			
Alone	490 (66.0)	250 (67.4)	240 (64.7)
With partner	156 (21.0)	89 (24.0)	67 (18.1)
With family/relative/friend	96 (12.9)	32 (8.6)	64 (17.3)
**On ARVs**			
Yes	727 (98.0)	366 (98.7)	361 (97.3)
**Time-dose adherence**			
Optimum *	555 (76.4)	283 (77.5)	272 (75.3)
**WHO stage of HIV [29]**			
Stage 1	359 (48.7)	183 (49.7)	176 (47.7)
Stage 2	139 (18.9)	74 (20.1)	65 (17.6)
Stage 3	174 (23.6)	76 (20.7)	98 (26.6)
Stage 4	64 (8.7)	34 (9.2)	30 (8.1)
**Condoms use since HIV diagnosis** ^μ^			
Always	268 (46.0)	143 (50.0)	125 (42.2)
Most of the time	68 (11.7)	26 (9.1)	42 (14.2)
Sometimes	72 (12.4)	47 (16.4)	25 (8.4)
Never	174 (29.9)	70 (24.5)	104 (35.1)
**Contraceptive use (excluding condoms)** ^μ^			
Used	364 (56.3)	174 (53.9)	190 (58.8)
**Any unintended pregnancy after HIV diagnosis** ^μ^			
Yes	45 (29.6)	15 (27.8)	30 (30.6)
**PMTCT utilization for pregnancy after HIV diagnosis** ^μ^			
Utilized service	106 (92.2)	31 (79.5)	75 (98.7)
**Social support (OSS-3)**			
Poor support	185 (25.2)	104 (28.2)	81 (22.2)
Intermediate support	284 (38.7)	128 (34.7)	156 (42.7)
Strong support	265 (36.1)	137 (37.1)	128 (35.1)
**Membership of any PLWH association (n = 736)**			
Member	62 (8.4)	39 (10.7)	23 (6.2)
**Perceived stigma**			
High perceived stigma	341 (46.8)	169 (46.4)	172 (47.1)
Low perceived stigma	388 (53.2)	195 (53.6)	193 (52.9)

ARV (Anti-retroviral), OSS-3 (Oslo Social Support– 3 items scale), HTC- HIV Testing and Counselling, PLWH (people living with HIV), PMTCT (Prevention of Mother to Child Transmission of HIV), WHO (World Health Organization)

* Optimum Adherence defined as taking ≥95% of prescribed ARV drugs within 2 hours of the prescribed time.

^μ^ Measure with most recent sexual partner

### Disclosure of HIV status

Of the 742 participants, 676 (91.1%) indicated that they had at least one sexual partner since their HIV diagnosis. Of the 676, 558 (82.5%) reported that they had disclosed their HIV status to their partner. However, of those who reported disclosing to their partners, 82 (14.7%) had at least one unprotected sexual encounter with the partner prior to disclosure; and of these, 10 (12.2%) did not know their partners status at the time of the study. The proportion of reported disclosure was higher among males, that is 291 (85.6%) of males compared to 267 (79.5%) of females. Table 3 describes the disclosure characteristics of participants.

**Table 3:** HIV status disclosure to most recent sexual partner and unprotected sex prior to disclosure, by gender.

Variable	Total	Male	Female
	N (%)	N (%)	N (%)
**Disclosure to partner** * **(n = 676)**			
Disclosed	558 (82.5)	291 (85.6)	267 (79.5)
Not disclosed	118 (17.5)	49 (14.4)	69 (20.5)
**Unprotected sex before disclosure to partner** * **(n = 558)**			
Yes	82 (14.7)	53 (18.2)	29 (10.9)
No	476 (85.3)	238 (81.8)	238 (89.1)

* Had unprotected sexual intercourse with most recent sexual partner after HIV diagnosis but prior to disclosure.

### Reasons for disclosure and non-disclosure

As can be seen from Table 4, among 558 participants who had disclosed their HIV status to their sexual partners, the commonest reasons reported for disclosure were: the need to get financial psychological or emotional support 206 (36.9%); not desiring to put partner at risk of catching HIV in 198 (35.5%); and encouragement from counsellors in 191 (34.2%) of cases. Among the 118 people who did not disclose their HIV status to their partner (s), “Fear of losing the relationship” was the commonest reason reported, among both males 22 (44.9%) and females 44 (63.8%). Other commonly reported reasons were “fear of being perceived as adulterous / unfaithful”, 21(17.8%) and “fear of verbal abuse” in 19 (16.1%) of cases.

**Table 4:** Reasons for disclosure / non-disclosure to most recent sexual partner by gender.

Variable	Total	Male	Female
	N (%)	N (%)	N (%)
Reasons* **for disclosing to sexual partner** ^μ^ **(n = 558)**			
To get support	206 (36.9)	124 (42.6)	82 (30.7)
Not to put partner at risk	198 (35.4)	122 (41.9)	76 (28.5)
Encouragement from counsellors	191 (34.2)	83 (28.5)	108 (40.4)
I don’t usually hold secrets from him/her	115 (20.6)	64 (22.0)	51 (19.1)
To initiate partner testing	65 (11.6)	16 (5.5)	49 (18.4)
Because tested together	61 (10.9)	31 (10.7)	30 (11.2)
Reasons* **for non-disclosure to sexual partner** ^μ^ **(n = 118)**			
Fear of losing the relationship	66 (55.9)	22 (44.9)	44 (63.8)
Fear of being perceived as adulterous / unfaithful	21(17.8)	8 (16.3)	13 (18.8)
Fear of verbal abuse	19 (16.1)	4 (8.2)	15 (21.7)
Saw no need to disclose	18 (15.3)	14 (28.6)	4 (5.8)
The person might be afraid of catching HIV from me	17 (14.4)	3 (6.1)	14 (20.3)
Fear of physical violence	14 (11.9)	0 (0)	14 (20.3)
Fear that the person may tell others	11 (9.3)	7 (14.3)	4 (5.8)
Not wanting to worry the person	8 (6.8)	3 (6.1)	(7.2)

*reporting of more than one reason possible

^**μ**^ Sexual partner = most recent sexual partner

### Process and outcomes of disclosure

Table 5 describes the initial outcome of disclosure to sexual partners. Of 558 participants who reported having disclosed to their partner, 511 (91.6%) used the process of active disclosure (i.e. disclosure initiated by the discloser), while 47 (8.4%) reported passive disclosure (i.e. disclosure only after a partner asked or partner found out from a third person).

With regards to the outcomes of 558 participants reporting disclosure, 380 (68.1%) reported receiving emotional/financial support, 378 (67.9%) reported that their disclosure led to discussions about safer sex, 281 (50.4%) were assisted to access HIV treatment, and 63 (11.3%) reported that their disclosure immediately initiated the partner to take an HIV test. However, 52 (9.3%) reported facing stigma and discrimination, 31 (5.6%) lost the relationship, and 24 (4.3%) faced verbal abuse. Physical violence was not reported as an outcome among either males or females; nonetheless, overall negative outcomes (stigma and discrimination, loss of a relationship as a direct outcome of the disclosure, and verbal abuse) were more common among females 70 (26.2%) compared to males 37 (12.7%).

**Table 5:** Processes and initial outcomes of disclosure to most recent sexual partner by gender.

Variable		Total	Male	Female
		N (%)	N (%)	N (%)
**Process of disclosure to partner** ^μ^				
Active	Self-initiated disclosure	492 (96.3)	259 (96.3)	233 (96.3)
	Assisted disclosure*	9 (1.8)	4 (1.5)	5 (2.1)
	Through a third person	10 (2.0)	6 (2.2)	4 (1.7)
	Total	511 (91.6)	269 (92.4)	242 (90.6)
Passive	Disclosure after partner asked	20 (42.6)	12 (54.5)	8 (32.0)
	Found out from others	27 (57.4)	10 (45.5)	17 (68.0)
	Total	47 (8.4)	22 (7.6)	25 (9.4)
**Initial outcomes of disclosure to sexual partner** ^μ^				
Emotional/financial support		380 (68.1)	205 (70.4)	175 (65.5)
Discussion about safer sex		378 (67.9)	212 (72.9)	166 (62.4)
Help to access HIV treatment		281 (50.4)	152 (52.2)	129 (48.3)
Cried (broke down emotionally)		166 (29.7)	110 (37.8)	56 (21.0)
Initiated partner’s HIV testing		63 (11.3)	19 (6.5)	44 (16.5)
Stigma and discrimination		52 (9.3)	9 (3.1)	43 (16.1)
Discontinuation of the relationship		31 (5.6)	14 (4.8)	17 (6.4)
Verbal abuse		24 (4.3)	14 (4.8)	10 (3.7)
Asked about past sexual history		13 (2.3)	6 (2.1)	7 (2.6)
Stayed neutral		6 (1.1)	3 (1.0)	(1.1)

* Assisted by family/friends/counsellor/other relatives

^μ^ Sexual partner = most recent sexual partner

### Factors associated with disclosure

As shown in Table 6, in the multiple regression model, disclosure was associated with greater condom use, greater social support, knowing the partner’s HIV status, having a good relationship with the partner, and cohabiting with the partner.

**Table 6:** Factors associated with HIV status disclosure to most recent sexual partner on multiple regression.

Variables	Disclosed N (%)	Not disclosed N (%)	Crude OR (95% CI)	Adjusted# OR (95% CI)
**Quality of relationship** ^**μ**^				
Mostly peaceful	459 (92.7)	36 (7.3)	10.56 (6.75–16.53)	4.16 (1.99–8.69) *
Mostly not peaceful	99 (54.7)	82 (45.3)	1.0	1.0
**Knowledge of partner’s status** ^**μ**^				
Known	506 (94.8)	28 (5.2)	30.93 (18.54–51.59)	15.02(7.29–30.94) *
Unknown	52 (36.9)	89 (63.1)	1.0	1.0
**Condom use with partner** ^**μ**^				
Always (100% of the time)	255 (95.1)	13 (4.9)	8.38 (4.566–15.38)	6.20 (2.52–15.25) *
Not always (<100%)	220 (70.1)	94 (29.9)	1.0	1.0
**Social support[30]**				
High	224 (90.0)	25 (10.0)	6.51 (3.88–10.91)	2.98 (1.09–8.14) *
Low	95 (57.9)	69 (42.1)	1.0	1.0
**Cohabitation status** ^**μ**^				
Living together	503 (87.8)	70 (12.2)	6.34 (3.95–10.17)	2.50 (1.13–5.53) *
Not living together	51 (53.1)	45 (46.9)	1.0	1.0

# Adjusted for socio-demographic, partnership, psycho-social, HIV and HIV care service related variables

* P < 0.05 = statistically significant

^μ^ Partner = most recent sexual partner.

## Discussion

This study has shown that most PLWH getting HIV care in public HIV clinics in Addis Ababa have disclosed their HIV status to their sexual partner(s); however, some had unprotected sexual encounter with the partner prior to disclosure. About a third of those who had unprotected sexual intercourse prior to disclosure did so while reckoning their partner’s HIV status to be negative. While some PLWH, particularly women, experience negative outcomes to disclosure such as stigma and discrimination; most often, disclosure was found to have positive outcomes such as initiating partner testing, obtaining psychological and/or financial support, and gaining support to access HIV care. However, some participants faced negative outcomes such as partner breaking down emotionally and stigma and discrimination. Additionally, disclosure was associated with greater condom use, greater social support, knowing the partner’s HIV status, having a good relationship with the partner, and cohabiting with the partner.

### Characteristics of participants

The study population closely reflected the age range most commonly affected by HIV, both globally [31] and locally [20], with the majority (84%) of participants, between the ages of 21 and 49 years. Similarly, the observation that 21 (5.6%) males and 61 (16.5%) females had no formal education, are consistent with the most recent data from the general population of Addis Ababa.Additionally, a significant proportion of participants (22.4%) were in serodiscordant relationships, which was also consistent withprevious findings from Addis Ababa [20].

### Disclosure of HIV status and serodiscordance

This study shows that most PLWH getting HIV care in public HIV clinics in Addis Ababa have disclosed their HIV status to their sexual partner(s). The overall disclosure level of 82.5%, was similar to that of a studies from Northern Ethiopia (76.6%) [32]; South Africa (80%) [33]; and a three country study in Tanzania, Kenya, and Namibia (80%) [34]. However, it is much higher than the 2006 figure of 60.5% from Addis Ababa [35].The significantly higher rate of disclosure in this study might be either because the 2006 study measured disclosure only as a secondary variable and/or because stigmatizing attitudes towards PLWH have declined significantly in Ethiopia over the past decade [36], related to improved access to HIV treatment, rendering HIV/AIDS a chronic manageable disease [20, 37].

Nevertheless; of the 558 (82.5%) of participants in this study who had disclosed to their partners at the time of the study, 82 (14.7%) reported having at least one unprotected sexual encounter prior to disclosure. Significant proportion (12.2%) of these did not know their partner’s status at the time of the study. This is a remarkable observation because a delay in disclosure may put partners at continued risk. Rates of delayed disclosure found in our study are similar to others reported from South-West Ethiopia (14.1%) [18] and Tanzania (15.1%) [38], but are lower than a report of 33.3% from the United States (US) [39]. This might be because, compared to Ethiopia and Tanzania, PLWH in developed nations such as the US have much higher access and utilization of effective HIV treatment, and subsequent viral suppression; and thus, might not feel they are putting their partner at a significant risk.

Overall, 93.0% of participants reported disclosure to at least one person, which is consistent with studies from South-West Ethiopia (94.5%) [18] and Tanzania (93.3%) [38]. Although the majority of participants had disclosed to at least one person, the 7% who had not disclosed to any one is particularly worrying, as they were effectively disconnected from any source of specific social support.

### Reasons for disclosure and non-disclosure

The commonest reasons reported for disclosure of HIV status to partners were: the need to get financial, psychological or emotional support (36.9%); not desiring to put partner at risk (35.5%), and encouragement from counsellors (34.2%).These findings are consistent with several other studies which were able to classify reasons for disclosure as “self-focused” (e.g. the need for support), “others-focused” (conveying a sense of responsibility) [18, 40, 41] [39, 42], and “counsellor’s advice” [38].

Remarkably, even if HIV non-disclosure to partners can potentially be prosecuted under the Ethiopian criminal code, none of the participants mentioned fear of prosecution as a reason for their decision to disclose despite its inclusion as one of the possible responses. This suggests that fear of prosecution might not be good enough to motivate disclosure. In support of this rationalization, other studies have found that, criminal HIV disclosure laws actually have detrimental effect -reducing HIV testing willingness [43] and diminishing the effectiveness of HIV prevention responses [44]. Indeed, with the wider availability of effective ART and with more people in Ethiopia becoming familiar with HIV transmission and safer sexual practice, it may be an opportune time to review the effectiveness of the current legal situation. However, another possible rationalization is that the counsellors in the clinics did not inform clients that non-disclosure can potentially lead to prosecution under the Ethiopian criminal code, and that providers are required to disclose their client’s HIV status to partners of persistent non-disclosers. This explanation is strengthened by the observation that only 27.5% of those who did not disclose reported that the counsellor had informed them of his/her obligation under the Ethiopian law. In light of this, there may be a need to familiarize HIV care providers with the country’s laws and the guidelines of the Ministry of Health (MOH).

Consistent with other studies from Uganda [45], Tanzania [38], and South-West Ethiopia [18], “Fear of losing the relationship” was the commonest reported reason for non-disclosure. Moreover, consistent with a hospital based study in South-West Ethiopia [46], there were gender differences in the reasons for non-disclosure to partners. A greater proportion of females, (21.7%), indicated that, “fear of verbal abuse” discouraged them from disclosing to their partners compared to males, (8.2%). Fear of physical violence was also reported more commonly as a reason for non-disclosure to partners among females, (20.3%), but not males (0%). This may be due to the high level of intimate partner violence against women in Ethiopia [6]. However, only 4.8% females and 3.7% of males in this study indicated that they experienced verbal abuse, and no participant reported experiencing physical abuse as a result of their disclosure.

### Process and outcomes of disclosure

Active disclosure of HIV status was more common than passive disclosure. Compared to those who passively disclosed, only a marginally higher proportion of participants who actively disclosed reported getting financial and psychological support—350 (68.5%) vs 30 (63.8%); Participants who actively disclosed were less likely to report loss of the relationship following active disclosure compared to those who reported passive disclosure (4.9% versus 12.8%). This observation was consistent with studies from Kenya [41] and Uganda [11]. This suggests that the outcomes of disclosure may vary with the mechanism used; although, more research is needed, preferably longitudinal, to determine the optimum processes and methods of disclosure most likely to yield a positive outcome for both disclosers and the confidantes.

The role of disclosure in HIV prevention strategies demonstrated by our observation that 11.3% of participants who disclosed reported that their partners subsequently went for HIV testing. Initiation of partner testing was also reported as a disclosure outcome by participants in study from Uganda [11]. Although the majority of participants indicated that they experienced a positive outcome following disclosure to a partner, a significant proportion were met with negative outcomes such as stigma, (9.3%); loss of the relationship (5.6%) and verbal abuse (4.3%). This finding is consistent with the 10.8% of 360 participants in a health-centre based study in North-East Ethiopia [47]; and 5% of 731 from South-West Ethiopia reporting adverse outcomes following disclosure. The latter study was limited to individuals who were sexually active at the time of the study with the partners to whom the disclosure rates were being recorded, thus may have not included individuals who had separated from their partners following negative response to disclosure, potentially leading to an underestimation of negative outcomes. Our study measured disclosure to the most recent partner, potentially allowing us to capture individuals who were separated from their partner following disclosure.

Females were five times more likely to report experiencing stigma following partner disclosure compared to males (16.1% versus 3.1%, respectively), possibly related to gender inequities and suggesting the need for gender-specific counselling. Fortunately, none reported physical abuse, an observation consistent with the finding of another hospital-based study from South-West Ethiopia [18].

Participants describing their relationships at the time of disclosure as “mostly peaceful” reported more positive outcomes to disclosure and fewer negative outcomes, compared to those describing their relationship as “mostly quarrelsome” (a direct translation of the Amharic term used in the questionnaire). This is consistent with a study from the US, which found that history of being in an abusive relationship was associated with experiencing verbal, physical and/or sexual abuse following disclosure [48].

### Factors associated with disclosure

In this study, participants who reported disclosure to their partner were over 6 times more likely to use condoms regularly with their partner, an observation consistent with many other studies [49] [50] [8]. However, this finding is markedly different from other Ethiopian studies, which found no evidence of association between disclosure and condom use [18, 32, 46, 47]. This discrepancy could be explained by the difference in study population. Unlike the other studies conducted in regional areas, this study was conducted in Addis Ababa; where there is higher literacy rate, awareness about HIV, and access to resources including condoms.

Disclosure was also found to have association with degree of social support. Those who disclosed to partners were three times more likely to report a greater level of social support, compared to those who had not disclosed, an observation consistent with some US studies [51, 52], but differing from observations from several other Ethiopian studies [8, 9, 18, 32, 46, 47]. Again, this might be explained by less prevalence of discriminatory attitudes towards PLWH in Addis Ababa compared to regional states where most of the other studies were undertaken. This observation may be of particular significance, as greater social support is associated with better psychological health, slower disease progression [53], and higher HIV care adherence [54]. Our study did not find any association between ART adherence and disclosure, but this may be due to reporting bias.

The other variables we found to be independent predictors of partner disclosure were: knowledge of partner’s HIV status; having a good relationship with their partner; and living with a partner. These observations are consistent with those of others from Uganda and Ethiopia, who found partner disclosure to be associated with knowledge of partner’s HIV status [11, 18, 47], having a good relationship with their partner [9, 47, 55], and living with a partner [11, 18].

## Conclusion and recommendation

HIV disclosure was common amongst individuals receiving HIV care in Addis Ababa; although sometimes delayed, with possible consequences for onward transmission. Moreover, a significant proportion of individuals continue to have unprotected sexual intercourse prior to disclosure, with partners whose HIV status they do not know. We recommend that HIV prevention efforts in Ethiopia focus on disclosure and safer sexual practice. This could be achieved by: [1] training HIV care providers to counsel clients about the potential benefits of disclosure to themselves and their partner(s), giving due emphasis to the needs and rights of partners to make informed decision on the level of risk they want to assume; [2] including disclosure counselling not only in the initial HIV client visit, as is the current practice, but also in follow-up sessions; and [3] including practical safer-sex education early in HIV care.

Disclosure was found to have mostly positive immediate outcomes such as getting financial and/or emotional support, as well as initiating partner testing. However; few percentages of PLWH, particularly women, experience negative outcomes such as stigma and discrimination. The major barriers to disclosure were found to be fear of negative outcomes such as losing the relationship and facing verbal/physical abuse. We recommend that HIV care providers and counsellors be trained to address this barrier by emphasising the positive outcomes of disclosure, while at the same time preparing their clients to handle a potential negative outcome. We also recommend that more researches (preferably longitudinal) be conducted to determine the optimum processes and methods of disclosure most likely to yield a positive outcome for both disclosers and the confidantes. The use of research-based algorithms could also assist with the targeting and support of individuals who are most at risk of experiencing negative reactions following disclosure.

Disclosure correlates with greater condom use and greater social support. Efforts to support disclosure have the potential to contribute to HIV control and prevention by encouraging safer sexual practice, promoting partner testing, and enhancing support for PLWH. In addition, disclosure also correlates with knowing the partner’s HIV status, having a good relationship with the partner, and cohabiting with the partner Therefore, we recommend targeting of disclosure counselling resources towards those who don’t know their partner’s HIV status, those who do not have a good relationship with their partner, and those who do not live with their partner.

### Strengths of the study

The large sample size (742) and the 100% response rate enabled sufficient power to investigate the multifactorial nature of disclosure and find several independent contributing factors. There was no direct benefit to participants, potentially reducing the likelihood of reporting bias. Non-disclosers and those who were found to have depression were referred for counselling and treatment respectively.

### Limitations of the study

Although fully informed, documented, consent was sought from all participants, and the voluntary nature of participation stressed, the 100% response rate may indicate a sense of obligation to comply, with the potential to influence responses. The use of a cross-sectional design meant that causality could not formally be established. Participants were investigated during their attendance at outpatient HIV clinic settings, where 98% were on antiretrovirals, and the generalisability to other PLWH is unknown. Furthermore, the reliance on self-reporting meant that the data were potentially subject to reporting and recall bias.

## Supporting information

S1 Questionnaire Questionnaire in English. (DOCX)

S2 Questionnaire Questionnaire in Amharic. (DOCX)
