# Supplementary material for: HIV Disclosure: HIV-positive status disclosure to sexual partners among individuals receiving HIV care in Addis Ababa, Ethiopia
Source: PLoS One. 2019 Feb 15;14(2):e0211967. doi: 10.1371/journal.pone.0211967 (PMC6415764; doi:10.1371/journal.pone.0211967)
Supplement: S1 Questionnaire — (DOCX) [file pone.0211967.s001.docx]

| 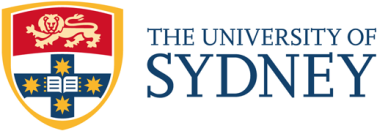 | | |  | **Discipline of HIV, STIs, and Sexual Health School of medicine Faculty of medicine** |
| --- | --- | --- | --- | --- |
|  | ABN 15 211 513 464 |  | | |
|  | **Richard Hillman**  *Associate professor* | Level 4  Jeffery House  The University of Sydney  NSW 2006 AUSTRALIA  Telephone: +61 2 9762 5386  Facsimile: +61 2 97625387  Email: [richard.hillman@sydney.edu.au](mailto:richard.hillman@sydney.edu.au)  Web: <http://www.sydney.edu.au/> medicine/wsshc | | |

**Questionnaire**

**A Questionnaire to study Outcomes of HIV Status Disclosure and associated factors in selected Public hospitals in Addis Ababa Ethiopia, 2015**, **University of Sydney**

| **Initials of name of interviewer:__________** | **Name of the Health Facility:_______________** |
| --- | --- |

| **Section I Demographic and Socioeconomic Information** | | | | | | | | | | | | | | | | | | | | |
| --- | --- | --- | --- | --- | --- | --- | --- | --- | --- | --- | --- | --- | --- | --- | --- | --- | --- | --- | --- | --- |
| **SN** | | | **Questions and Filters** | **Response & Coding Categories** | | | | | | | | | | | | | | **Skip** | | |
| 101 | | | Study Number | ________________ | | | | | | | | | | | | | |  | | |
| 102 | | | First two letters of first name and first two letters of last name | _______ _______ | | | | | | | | | | | | | |  | | |
| 103 | | | Sex *Check box (✓)* | 🞏 1 = Male 🞏 2 = Female | | | | | | | | | | | | | |  | | |
| 104 | | | How old are you? | ______________ Years | | | | | | | | | | | | | |  | | |
| 105 | | | Are you able to read and write in any language? | 🞏 1 = Yes 🞏 2 = No | | | | | | | | | | | | | | Skip to Q. 107 | | |
| 106 | | | If said yes above, have you ever attended formal school? | 🞏 1 = Yes (level _______ ) 🞏 2 = No | | | | | | | | | | | | | |  | | |
| 107 | | | What is your religious denomination?  Check box (✓) | 🞏 1 = Orthodox  🞏 2 = Muslim  🞏 3 = Protestant | | | | | 🞏 4 = Catholic  🞏 5 = Other, Specify _____________________ | | | | | | | | |  | | |
| 108 | | | What is your ethnic group? *Check box (✓)* | 🞏 1 = Oromo  🞏 2 = Amhara  🞏 3 = Tigre | | | | | 🞏 4 = Guraghe  🞏 5 = Other, Specify __  __________________ | | | | | | | | |  | | |
| 109 | | | What is your major paid occupation currently?  (Whatever you do to earn money)? | 🞏 1 = Government employee  🞏 2 = Private/ Non-government organization employee  🞏 3 = Own business/farm  🞏 4 = Daily laborer | | | | | 🞏 5 = Housewife  🞏 6 = Student  🞏 7 = Have no Job  🞏 8 = Other specify __________________ | | | | | | | | |  | | |
| 110 | | | How much is your household income, including your own? | | | | | | ___________(Birr/month) | | | | | | | | |  | | |
| 111 | | | Which of the following items do you have in your household? (Wealth Index) | | | | | | | | | | | | | | |  | | |
|  | | | Electricity | Yes | | | | | No | | | | | | | | |  | | |
|  |  |  | Clock | Yes | | | | | No | | | | | | | | |  |  |  |
|  |  |  | Radio | Yes | | | | | No | | | | | | | | |  |  |  |
|  |  |  | Television | Yes | | | | | No | | | | | | | | |  |  |  |
|  |  |  | Mobile Telephone | Yes | | | | | No | | | | | | | | |  |  |  |
|  |  |  | Refrigerator | Yes | | | | | No | | | | | | | | |  |  |  |
|  |  |  | Separate room for kitchen | Yes | | | | | No | | | | | | | | |  |  |  |
|  |  |  | Bicycle | Yes | | | | | No | | | | | | | | |  |  |  |
|  |  |  | Savings Bank Account | Yes | | | | | No | | | | | | | | |  |  |  |
|  |  |  | Toilet | Yes | | | | | No | | | | | | | | |  |  |  |
|  |  |  | Type of floor | Mud/soil | | | | | Cement/wood | | | | | | | | |  |  |  |
|  | | | Type of wall | No wall/only mud | | | | | Cement/wood/plaster with mud | | | | | | | | |  | | |
| 112 | | | What would you say is your family’s main source of income to cover major expenses (school fees, food, health care, house rent...)? | 🞏 1= your own earnings  🞏 2 = Spouse’s earnings or support from his/her family | | | | | 🞏 3 = Support from your family of origin  🞏 4 = Other, specify ________________ | | | | | | | | |  | | |
| 113 | | | Which of these substances do you use currently, if any? | 🞏 1= Khat  🞏 2 = cigarette  🞏 3 = Alcohol | | | | | 🞏 4 = Other (specify)  ___________________  🞏 5 = I don’t use any | | | | | | | | |  | | |
| **Section II Partnership information.** Now, I am going to ask you questions regarding your relationship with your most recent sexual partner. Your most recent partner is the person with whom you, most recently, are having/had mostly sex with. If you are abstaining, please answer about your spouse. Regular partner is a person with who you have sex regularly but isn’t your spouse. Casual partner is a person with whom you had sex only once or only infrequently. If you are mostly having casual partners recently, please answer about your most recent casual partner. | | | | | | | | | | | | | | | | | | | | |
| 201 | Have you ever had sexual partner since knowing your HIV status? | | | 🞏 1 = yes | | | | | | 🞏 = 2 No | | | Skip all questions about partnership | | | | | | | |
| 202 | What is/was your relationship status with your most recent sexual partner?  *Check box (✓)* | | | 🞏 1= Married  🞏 2=Unmarried (has regular boy /girlfriend) | | | | | | 🞏 3= Unmarried ( has casual sexual partner (s))  🞏 4 = Unmarried (never had sexual partner) | | | | | | | | | Skip to Q 301 | |
| 203 | How is/was your relation with your most recent partner? | | | 🞏 1 = We are/were living together | | | | | | 🞏 2 = Not living together | | | | | | | | |  | |
| 204 | For how long do you/did you know your partner? | | | _________________________________________ | | | | | | | | | | | | | | |  | |
| 205 | How long do you/did you think this relationship will last? | | | 🞏 1 = For the rest of your life  🞏 2 = For a long while | | | | | | 🞏 3 = For a while ,but not too long  🞏 4 = Only a short while | | | | | | | | |  | |
| 206 | How old is/was your most recent partner? | | | ______________________ | | | | | | | | | | | | | | |  | |
| 207 | Is/was your most recent partner able to read and write in any language | | | 🞏 1 = Yes | | | | | | 🞏 2 = No | | | | | | | | | Skip to Q. 209 | |
| 208 | If the response for the above question is yes, have he/she ever attended formal school? | | | 🞏 1 = Yes ( Grade ___________ ) | | | | | | 🞏 2 = No | | | | | | | | |  | |
| 209 | How do you see the relation with your most recent partner (*ask until disclosure, if disclosed*)? | | | 🞏 1 = peace full  🞏 2 = We quarreled some times  🞏 3 = We often quarreled | | | | | | 🞏 4 = We often quarreled and have (are going to) separate(d)  🞏 5 = Other specify ___________ | | | | | | | | |  | |
| 210 | Have you ever talked about HIV testing with your partner before going for a test? | | | 🞏 1 = Yes | | | | | | 🞏 2 = No | | | | | | | | | Skip to Q 212 | |
| 211 | What was your partner’s intention about the discussion on HIV testing? | | | **🞏** 1= Was happy  **🞏** 2= Was reluctant to discuss | | | | | | **🞏** 3= Got angry  **🞏** 4 = Other | | | | | | | | |  | |
| 212 | Is your most recent partner tested for HIV, what was the result? | | | **🞏** 1=Has tested positive  **🞏** 2=Has tested negative  **🞏** 3= Has tested, but I don’t know the result | | | | | | **🞏** 4= Hasn’t tested  **🞏** 5= I don’t know if he/she is tested | | | | | | | | |  | |
| 213 | If you didn’t know, what do you think about your partner’s HIV status? | | | **🞏** 1= HIV positive  **🞏** 2= HIV negative | | | | | | **🞏** 3 = I can’t tell | | | | | | | | |  | |
| **Section III HIV testing and treatment** | | | | | | | | | | | | | | | | | | | | |
| 301 | What were the main reasons Why you went for HIV testing  (more than one answer possible) | | | **🞏** 1=Your partner told you he/she is HIV positive  **🞏** 2=Your own past sexual behavior  **🞏** 3=Your partner’s past sexual behavior  **🞏** 4=Your partner told you to come in and get tested  **🞏** 5=Your partner at the time was sick or died  **🞏** 6=You had sharp object injury or blood transfusion  **🞏** 7=You were taking care of people with HIV/AIDS | | | | | | **🞏** 8=You just wanted to know your HIV status  **🞏** 9=You were sick  **🞏** 10=workplace / embassy requires testing  **🞏** 11=You were planning to get married  **🞏** 12=You were planning on having children  **🞏** 13= You were pregnant so you cared about the fetus  **🞏** 14=Other, specify | | | | | | | | |  | |
| 302 | How long had you been thinking about coming in for HIV testing before you decided to be tested? | | | **🞏** 1. Days _______  **🞏** 2. Weeks _______  **🞏** 3.Months _______ | | | | | | **🞏** 4. Years _________  **🞏** 5. Other, specify:________________ | | | | | | | | |  | |
| 303 | When were you diagnosed with HIV? | | | ­­____________________________ (month, year) | | | | | | | | | | | | | | |  | |
| 304 | Were you counseled before or after test? | | | 🞏 1 = Yes | | | | | | 🞏 2 = No | | | | | | | | |  | |
| 305 | With whom did you test for HIV | | | 🞏 1= Alone  🞏 2= With my partner | | | | | | 🞏 3 = Other specify__________________ | | | | | | | | |  | |
| 306 | Did the counselor talk to you about disclosing your HIV positivity to your partner, family, or significant others? | | | 🞏 1 = Yes | | | | | | 🞏 2 = No | | | | | | | | |  | |
| 307 | Did the counselor tell you that he/she is required to notify your partner s/he is at risk, if you wouldn’t? | | | 🞏 1 = Yes | | | | | | 🞏 2 = No | | | | | | | | |  | |
| 308 | Are you on antiretroviral medication? | | | 🞏 1 = Yes,  **Regimen** _________  **How long** ___ months/yrs | | | | | | 🞏 2 = No | | | | | | | | | Skip to Q 312 | |
| 309 | How many times have you missed taking your drugs in the past one month? (Do pill count) | | |  | | | | | | | | | | | | | | |  | |
| 310 | How many times in the past one month have you failed to take the medication within 2 hours of the proper time? | | | ________________ | | | | | | | | | | | | | | |  | |
| 311 | If you missed a dose or medication time in the past one month, how often was “being with others who did not know you were HIV-positive and not wanting them to notice you taking medication”, the reason for the miss? | | | 🞏 1 = Never  🞏 2 = Sometimes  🞏 3 = Often | | | | | | | | | | | | | | |  | |
| 312 | Are you a member of any association of PLWH? | | | 🞏 1 = Yes | | | | | | 🞏 2 = No | | | | | | | | |  | |
| 313 | Stage of the disease (look to patient card) | | | 🞏 1 = Stage I  🞏 2 = Stage II  🞏 3 = Stage III | | | | | | 🞏 4 = Stage IV  🞏 5 = Not specified | | | | | | | | |  | |
| **Section IV. Disclosure out comes and barriers (remember to tick BOTH boxes)** | | | | | | | | | | | | | | | | | | | | |
| 401 | Does anyone close to you (other than the diagnosing / treating health professional) know your HIV status? | | | 🞏 1 = Yes | | | | | | 🞏 2 = No | | | | | | | | | Skip to Q.411 | |
| 402 | If yes, who knows that you are HIV positive?  (Multiple response possible) | | | 🞏 1= Most recent partner  🞏 2 = Mother  🞏 3 = Father  🞏 4 = Son/Daughter  🞏 5 = Sibling  🞏 6 = Other Family members | | | | | | 🞏 7= Relative  🞏 8 = Friend  🞏 9 = Religious Leader  🞏 10 = Neighbor  🞏 11 = My usual Doctor / Nurse / Health Officer  🞏 12 = Others | | | | | | | | |  | |
| 403 | How did your **most recent partner** (tick first box) and the **first person from your** **family / close relatives** to know your HIV positivity (thick second box) know your HIV positivity? | | | 🞏 🞏 1 = You disclosed alone / by yourself  🞏 🞏 2 = You disclosed with another person  🞏 🞏 3 = You made other people to tell him/her | | | | | | 🞏 🞏 4 = he/she saw your medicines / symptoms / test paper… and asked; then you told it  🞏 🞏 5 = he/she found out from other people | | | | | | | | |  | |
| 404 | How long after diagnosis or after you started relationship (if you had already been tested positive when you started the relationship) did you tell your result to your most recent partner? | | | ____________________________________  (specify days, weeks, months, years as much as possible) | | | | | | | | | | | | | | |  | |
| 405 | How long after diagnosis did you tell the first time your result to your family / close relative? | | | ____________________________________  (specify days, weeks, months, years as much as possible) | | | | | | | | | | | | | | |  | |
| 406 | After you found out that you are HIV positive, did you have unprotected sex with your partner before telling him/her about your infection? | | | 🞏 1 = Yes | | | | | | 🞏 2 = No | | | | | | | | |  | |
| 407 | Why did you tell to your most recent partner (tick first box) and the first of your family/close relative to hear it from you (tick second box) that you are HIV positive?  (Multiple response possible) | | | 🞏 🞏 1 = The counsellor encouraged me  🞏 🞏 2 = I do not want to put them at risk  🞏 🞏 3 = I want to get their support  🞏 🞏 4 = It is usual to tell them every secret things | | | | | | 🞏 🞏 5 = I do not want to be legally accused  🞏 🞏 6 = I feared God to hide such things  🞏 🞏 7 = others(specify) ___________________ | | | | | | | | |  | |
| 408 | How did your most recent partner (tick first box) and family/close relative (tick second box) react when they heard that you are HIV positive?  (Multiple response possible) | | | 🞏 🞏1= Supported me (emotional, financial)  🞏 🞏2= broke down emotionally (confusion /sadness /anger)  🞏 🞏3 = I got freedom to access HIV treatment / follow-up  🞏 🞏4 = physical violence  🞏 🞏 5 = verbal violence or threatening  🞏 🞏 6 = inflicted stigma and discrimination on me | | | | | | 🞏 🞏 7 = Worried about his/her own HIV status  🞏 🞏 8 = Asked about my sexual history  🞏 🞏 9 = Rejected/stopped our relationship  🞏 🞏 10 = Other (specify) ___________________  ___________________ | | | | | | | | |  | |
| 409 | How would you compare the effect on you of the immediate vs current reactions of your most recent partner (tick first box) and first family/close relative disclosed to (tick second box)? | | | 🞏 🞏 1 = first helpful – later helpful  🞏 🞏 2 = First helpful – later unhelpful | | | | | | 🞏 🞏 3 = First unhelpful – later helpful  🞏 🞏 4 = First unhelpful – later unhelpful | | | | | | | | |  | |
| 410 | Did you and your most recent sexual partner discus about safer-sex after your HIV status was disclosed? | | | 🞏 1 = Yes | | | | | | 🞏 2 = No | | | | | | | | |  | |
| 411 | If you have NOT told your HIV status to your partner (tick first box) family / close relative (tick second box) what is your reason?  (Multiple response possible) | | | 🞏 🞏1=The person might stop our relationship  🞏 🞏 2=The person might be afraid of catching HIV from me  🞏 🞏 3= The person might hurt/ threaten me verbally  🞏 🞏 4 = The person is too young to handle it  🞏 🞏 5 = The person may tell others  🞏 🞏 6 = The person had too many other problems to deal with right now | | | | | | 🞏 🞏 7 = There was no need to tell  🞏 🞏 8 = I do not want to worry him/her  🞏 🞏 9 =The person might hurt me physically  🞏 🞏 10 = The person might murder me  🞏 🞏 11 = The person might think I am adulterous / unfaithful  🞏 🞏 12 = Other (Specify) _____________________  _____________________ | | | | | | | | |  | |
| **Section V. Sexual behavior and experience** | | | | | | | | | | | | | | | | | | | | |
| 501 | How many sexual partners have you had in your life time? | | | _____________________ | | | | | | | | | | | | | | |  | |
| 502 | How many sexual partners do you have currently? | | | ____________________ | | | | | | | | | | | | | | |  | |
| 503 | How often do you use condom with your most recent sexual partner since you have been diagnosed HIV positive? | | | 🞏 1 = Always (100% of the time)  🞏 2 = Most of the time  🞏 3 = Sometimes | | | | | | 🞏 4 = I do not use at all  🞏 5 = abstained | | | | | | | | |  | |
| 504 | If you are not using condom 100% of the time, what is your reason for that?  (Multiple response possible) | | | 🞏 1 = She/he is HIV positive  🞏 2 = Condoms reduce pleasure  🞏 3 = Condoms would make my partner suspicious of my positive status  🞏 4 = Found it difficult to use / put on  🞏 5 = Found it difficult to discuss condoms  🞏 6 = Could not obtain them / did not have them with me  🞏 7 = Other (Specify)____________________ | | | | | | | | | | | | | | |  | |
| 505 | What form of birth control are you using with your most recent partner?  (You can give more than one answer) | | | 🞏 1 = Not using any method  🞏 2 = pill  🞏 3 = Contraceptive injection  🞏 4 = Norplant | | | | | | 🞏 5 = Diaphragm  🞏 6 = Use condoms  🞏 7 = Sterilization  🞏 8 = Other (Specify) ______________________ | | | | | | | | |  | |
| 506 | If not using any of the methods, why is your reason? | | | 🞏 1 = We practice(d) abstinence  🞏 2 = we wanted to have a child | | | | | | 🞏 3= Other (Specify) __________________________________________________________________ | | | | | | | | |  | |
| **NON- REGULAR PARTNERS *(ONLY for those whose most recent partner is/was their spouse or regular partner)*** Now I would like to ask you some questions about your ***non-regular partners*** Sexual partners that you are not married to and never lived with. **By non-regular I mean someone with whom you have had sex only once or very rarely**. | | | | | | | | | | | | | | | | | | | | |
| 507 | Have you had sex with a non-regular partner (s) in the past 1 year? | | | 🞏 1 = Yes | | | | | | 🞏 2 = No | | | | | | | | | Skip to Q 601 | |
| 508 | What is your most recent non-regular partner’s HIV status? | | | 🞏 1 = HIV positive  🞏 2 = HIV negative | | | | | | 🞏 3 = I don’t know | | | | | | | | |  | |
| 509 | If “I don’t know”, what do you think is your most recent non-regular partner’s HIV status? | | | 🞏 1 = HIV positive  🞏 2 = HIV negative | | | | | | 🞏 3 = I can’t tell | | | | | | | | |  | |
| 510 | Does your most recent non-regular partner know your HIV status? | | | 🞏 1 = yes | | | | | | 🞏 2 = No | | | | | | | | | Skip to Q512 | |
| 511 | If yes, how did he/she knew? | | | 🞏 1 = you told him/her  🞏 2 = he/she found out from other people | | | | | | 🞏 3 = he/she saw your medicines / symptoms / test paper… and asked; then you told it | | | | | | | | |  | |
| 512 | How often do you use condom with your most recent non-regular partner? | | | 🞏 1 = Always (100% of the time)  🞏 2 = Most of the time | | | | | | 🞏 3 = Sometimes  🞏 4 = We do not use at all | | | | | | | | |  | |
| **Section VI. Social Support (OSLO SOCIAL SUPPORT SCALE)** | | | | | | | | | | | | | | | | | | | | |
| 601 | How many people are so close to you that you can count on them if you have serious problems? | | | None | | | | | | | | | | 1 | | | | |  | |
|  |  |  |  | 1 or 2 | | | | | | | | | | 2 | | | | |  |  |
|  |  |  |  | 3-5 | | | | | | | | | | 3 | | | | |  |  |
|  |  |  |  | 6 or more | | | | | | | | | | 4 | | | | |  |  |
| 602 | How much concern do people show in what you are doing? | | | A lot of concern and interest | | | | | | | | | | 5 | | | | |  |  |
|  |  |  |  | Some concern and interest | | | | | | | | | | 4 | | | | |  |  |
|  |  |  |  | Uncertain | | | | | | | | | | 3 | | | | |  |  |
|  |  |  |  | Little concern and interest | | | | | | | | | | 2 | | | | |  |  |
|  |  |  |  | No concern and interest | | | | | | | | | | 1 | | | | |  |  |
| 603 | How easy can you get practical help from neighbors if you should need it? | | | Very easy | | | | | | | | | | 5 | | | | |  |  |
|  |  |  |  | Easy | | | | | | | | | | 4 | | | | |  |  |
|  |  |  |  | Possible | | | | | | | | | | 3 | | | | |  |  |
|  |  |  |  | Difficult | | | | | | | | | | 2 | | | | |  |  |
|  |  |  |  | Very difficult | | | | | | | | | | 1 | | | | |  |  |
| **Section VII Depression scale (Patient Health Questionnaire 9) Over the last two weeks** how often have you been bothered by any of the following problems? | | | | | | | | | | | | | | | | | | | | |
|  |  | | | | Not at all | | | Several days | | | | More than half the days | | | Nearly every day | | | | |  |
| 702 | Little interest or pleasure in doing things | | | | 0 | | | 1 | | | | 2 | | | 3 | | | | |  |
| 703 | Felling down, depressed, or hopeless | | | | 0 | | | 1 | | | | 2 | | | 3 | | | | |  |
| 704 | Trouble falling or staying asleep, or sleeping too much | | | | 0 | | | 1 | | | | 2 | | | 3 | | | | |  |
| 705 | Feeling tired or having little energy | | | | 0 | | | 1 | | | | 2 | | | 3 | | | | |  |
| 706 | Poor appetite or over eating | | | | 0 | | | 1 | | | | 2 | | | 3 | | | | |  |
| 707 | Feeling bad about yourself – or that you are a failure or have let yourself or your family down | | | | 0 | | | 1 | | | | 2 | | | 3 | | | | |  |
| 708 | Trouble concentrating on things such as reading the newspaper or watching television | | | | 0 | | | 1 | | | | 2 | | | 3 | | | | |  |
| 709 | Moving or speaking so slowly that other people could have notice or the opposite – being so fidgety or restless that you have been moving around a lot more than usual | | | | 0 | | | 1 | | | | 2 | | | 3 | | | | |  |
| 710 | Thought that you would be better off dead, or of hurting yourself | | | | 0 | | | 1 | | | | 2 | | | 3 | | | | |  |
|  |  | | | | **Add column** | | |  | | | |  | | |  | | | | |  |
|  |  | | | | **Total** | | |  | | | | | | | | | | | |  |
| 711 | If you checked off any problems, how difficult have these problems made it for you to do your work, take care of things at home, or get along with other people? (*Tick*) | | | | 🞏 1= Not difficult at all | | | | | | | 🞏 2= Somewhat difficult | | | | | | | |  |
|  |  |  |  |  | 🞏 3= Very difficult | | | | | | | 🞏4= Extremely difficult | | | | | | | |  |
| **VIII. Perceived stigma and discrimination** | | | | | | | | | | | | | | | | | | | | |
| This set of questions asks about some of your feelings and opinions as to how people with HIV feel and how they are treated. There is no right or wrong answer. Please feel free to tell us what you think. Could you tell me if you agree or disagree with the following statements | | | | | | | | | | | | | | | | | | | | |
|  |  | | | | | | Strongly disagree | | | | Disagree | | | Agree | | Strongly agree | | | | |
| 801 | In many areas of my life, no one knows that I have HIV | | | | | | 1=🞏 | | | | 2=🞏 | | | 3=🞏 | | 4=🞏 | | | | |
| 802 | I feel guilty because I have HIV | | | | | | 1=🞏 | | | | 2=🞏 | | | 3=🞏 | | 4=🞏 | | | | |
| 803 | People's attitudes about HIV make me feel worse about myself | | | | | | 1=🞏 | | | | 2=🞏 | | | 3=🞏 | | 4=🞏 | | | | |
| 804 | Telling someone I have HIV is risky | | | | | | 1=🞏 | | | | 2=🞏 | | | 3=🞏 | | 4=🞏 | | | | |
| 805 | People with HIV lose their jobs when their employers find out | | | | | | 1=🞏 | | | | 2=🞏 | | | 3=🞏 | | 4=🞏 | | | | |
| 806 | I work hard to keep my HIV a secret | | | | | | 1=🞏 | | | | 2=🞏 | | | 3=🞏 | | 4=🞏 | | | | |
| 807 | I feel I am not as good a person as others because I have HIV | | | | | | 1=🞏 | | | | 2=🞏 | | | 3=🞏 | | 4=🞏 | | | | |
| 808 | I never feel ashamed of having HIV | | | | | | 4=🞏 | | | | 3=🞏 | | | 2=🞏 | | 1=🞏 | | | | |
| 809 | People with HIV are treated like outcasts | | | | | | 1=🞏 | | | | 2=🞏 | | | 3=🞏 | | 4=🞏 | | | | |
| 810 | Most people believe that a person who has HIV is dirty | | | | | | 1=🞏 | | | | 2=🞏 | | | 3=🞏 | | 4=🞏 | | | | |
| 811 | It is easier to avoid new friendships than worry about telling someone that I have HIV | | | | | | 1=🞏 | | | | 2=🞏 | | | 3=🞏 | | 4=🞏 | | | | |
| 812 | Having HIV makes me feel unclean | | | | | | 1=🞏 | | | | 2=🞏 | | | 3=🞏 | | 4=🞏 | | | | |
| 813 | Since learning I have HIV, I feel set apart and isolated from the rest of the world | | | | | | 1=🞏 | | | | 2=🞏 | | | 3=🞏 | | 4=🞏 | | | | |
| 814 | Most people think that a person with HIV is disgusting | | | | | | 1=🞏 | | | | 2=🞏 | | | 3=🞏 | | 4=🞏 | | | | |
| 815 | Having HIV makes me feel that I'm a bad person | | | | | | 1=🞏 | | | | 2=🞏 | | | 3=🞏 | | 4=🞏 | | | | |
| 816 | Most people with HIV are rejected when others find out | | | | | | 1=🞏 | | | | 2=🞏 | | | 3=🞏 | | 4=🞏 | | | | |
| 817 | I am very careful who I tell that I have HIV | | | | | | 1=🞏 | | | | 2=🞏 | | | 3=🞏 | | 4=🞏 | | | | |
| 818 | Some people who know I have HIV have grown more distant | | | | | | 1=🞏 | | | | 2=🞏 | | | 3=🞏 | | 4=🞏 | | | | |
| 819 | Since learning I have HIV, I worry about people discriminating against me | | | | | | 1=🞏 | | | | 2=🞏 | | | 3=🞏 | | 4=🞏 | | | | |
| 820 | Most people are uncomfortable around someone | | | | | | 1=🞏 | | | | 2=🞏 | | | 3=🞏 | | 4=🞏 | | | | |
| 821 | I never feel the need to hide the fact that I have HIV | | | | | | 4=🞏 | | | | 3=🞏 | | | 2=🞏 | | 1=🞏 | | | | |
| 822 | I worry that people may judge me when they learn I have HIV | | | | | | 1=🞏 | | | | 2=🞏 | | | 3=🞏 | | 4=🞏 | | | | |
| 823 | Having HIV in my body is disgusting to me | | | | | | 1=🞏 | | | | 2=🞏 | | | 3=🞏 | | 4=🞏 | | | | |
| **IX. Perceived barriers & Self efficacy to disclosure** | | | | | | | | | | | | | | | | | | | | |
|  | |  | | | | | Strongly disagree | | | | Disagree | | | Agree | | Strongly agree | | | | |
| 901 | | If I disclose my HIV result I might suffer from stigma and discriminated | | | | | 1=🞏 | | | | 2=🞏 | | | 3=🞏 | | 4=🞏 | | | | |
| 902 | | If I disclose my HIV result my partner/family will leave/reject me | | | | | 1=🞏 | | | | 2=🞏 | | | 3=🞏 | | 4=🞏 | | | | |
| 903 | | I am certain that I can disclose my result Even if I face discrimination | | | | | 1=🞏 | | | | 2=🞏 | | | 3=🞏 | | 4=🞏 | | | | |
| 904 | | I am certain that I can disclose my result Even if my partner/family leave/reject me | | | | | 1=🞏 | | | | 2=🞏 | | | 3=🞏 | | 4=🞏 | | | | |
| X. Future intention | | | | | | | | | | | | | | | | | | | | |
| 1001 | | Do you plan to disclose your result to partner, family, or other significant people in your life? | | | | 🞏 1 = Yes | | | | | | 🞏 2 = No | | | | |  | | | |
| XI. PMTCT service (now I’ll ask you about pregnancies only after you/your partner tested HIV positive) | | | | | | | | | | | | | | | | | | | | |
| 1101 | | Have you/your most recent partner had conceived after one of you learnt you are HIV positive? | | | | 🞏 1 = Yes (how many_____) | | | | | | 🞏 2 = No | | | | | Finish here | | | |
| 1102 | | If yes, was it before or during the pregnancy that you/your partner learnt your positive HIV status? | | | | 🞏 1 = Before pregnancy | | | | | | 🞏 2 = During pregnancy | | | | |  | | | |
| 1103 | | Were any of the pregnancies unintended? | | | | 🞏 1 = Yes | | | | | | 🞏 2 = No | | | | |  | | | |
| 1104 | | Did you/ your pregnant partner go for PMTCT? (If more than one, ask about the first one) | | | | 🞏 1 = Yes | | | | | | 🞏 2 = No | | | | |  | | | |
| 1105 | | Did you/your pregnant partner and the newborn use ARVs? | | | | 🞏 1 = Yes | | | | | | 🞏 2 = No | | | | |  | | | |
| 1106 | | Did you/your partner breast-feed? | | | | 🞏 1 = Yes | | | | | | 🞏 2 = No | | | | |  | | | |

**THE END.**

**Thank you very much!**
