# Supplementary material for: HIV Disclosure: HIV-positive status disclosure to sexual partners among individuals receiving HIV care in Addis Ababa, Ethiopia
Source: PLoS One. 2019 Feb 15;14(2):e0211967. doi: 10.1371/journal.pone.0211967 (PMC6415764; doi:10.1371/journal.pone.0211967)
Supplement: S2 Questionnaire — (DOCX) [file pone.0211967.s002.docx]

| 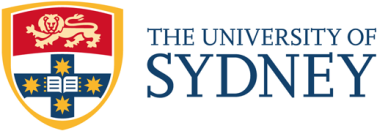 | |  | | የሲድኒ ዩኒቨርሲቲ  የኤችአይቪ፣አባለዘር እና የጾታ ጤና  ህክምና ት/ቤት  ህክምና ፋኩሊቲ |
| --- | --- | --- | --- | --- |
|  | |  | |  |
|  | ኤቢኤን 15 211 513 464 | | |  |
|  | **ሪቻርድ ሂልማን**  *ረዳት ፐሮፌሰር* | | ደረጃ 4  ጄፍሪ ሀውስ  የሲድኒ ዩኒቨርሲቲ  ኤንኤስደብሊው 2006 አውስትራሊያ  ስልክ +61 2 9762 5386  ፋክስ +61 2 97625387  ኢ-ሜይል [Richard.hillman@sydney.edu.au](mailto:Richard.hillman@sydney.edu.au)  ድረ ገጽ<http://www.sydney.edu.au/medicine/wsshc> | |

***መጠይቅ***

**የኤችአይ ቪ ኤድስ በደም ውስጥ መኖሩን ለሌሎች መግለፅ የሚያስከትለው ውጤትና ተያያዥ የሆኑ ጉዳዮችን በአዲስ አበባ ኢትዮጵያ በተመረጡ የመንግስት ሆስፒታሎች ውስጥ ለማጥናት የተዘጋጀ መጠይቅ፣ ሲድኒ ዩኒቨርሲቲ፣2015እኤአ**

| የቃለ መጠይቅ አድራጊው የመጀመሪያ ፊደላት፡ _________________ | የጤና ተቋሙ ስም፡ ______________ |
| --- | --- |

| **ክፍል 1 የስነ ህዝባዊና ማህበረ- መጣኔ ሀብታዊ መረጃ** | | | | | | | | | | | | | | | | | | | | | | |
| --- | --- | --- | --- | --- | --- | --- | --- | --- | --- | --- | --- | --- | --- | --- | --- | --- | --- | --- | --- | --- | --- | --- |
| ተ/ቁ | | ጥያቄና ማጣሪያ | | | ምላሽና የኮድ ክፍፍል | | | | | | | | | | | | | | | | ይለፉ | |
| 101 | | የጥናቱ ቁጥር | | | ______________________ | | | | | | | | | | | | | | | |  | |
| 102 | | የመጀመሪያው ስም የመጀመሪያዎቹ 2 ፊደላትና የመጨሻው ስም የመጀመሪያዎቹ 2 ፊደላት | | | ________ _________ | | | | | | | | | | | | | | | |  | |
| 103 | | ፆታ (ሳጥን ውስጥ የ√ ምልክት ያድርጉ) | | | 🞏1=ወንድ | | | | | | | | 🞏2=ሴት | | | | | | | |  | |
| 104 | | ዕድሜዎት ስንት ነው ? | | | _______ አመት | | | | | | | | | | | | | | | |  | |
| 105 | | በማንኛውም ቋንቋ ማንበብና መፃፍ ይችላሉ? | | | 🞏1= አዎ | | | | | | | | 🞏2= የለም | | | | | | | | ወደጥያቄ 107ይለፉ | |
| 106 | | ከላይ ላለው ጥያቄ መልስዎ አዎ ከሆነ መደበኛ ትምህርት ተምረዋል | | | 🞏1=አዎ(ደረጃ ________) | | | | | | | | 🞏2= የለም | | | | | | | |  | |
| 107 | | ሃይማንዎትዎ ምንድነው ? (ሳጥን ውስጥ √ ያድርጉ) | | | 🞏1= ኦርቶዶክስ 🞏2= ሙስሊም 🞏3= ፕሮቴስታንት | | | | | | | 🞏4= ካቶሊክ  🞏5= ሌላ ከሆነ ይግለፁ ________ | | | | | | | | |  | |
| 108 | | ብሔርዎ ምንድ ነው? (ሳጥን ውስጥ √ ያድርጉ) | | | 🞏1= ኦሮሞ  🞏2= አማራ  🞏3= ትግሬ | | | | | | | 🞏4= ጉራጌ  🞏5= ሌላ ከሆነ ይግለፁ ________ | | | | | | | | |  | |
| 109 | | በአሁኑ ጊዜ በዋናነት ተከፋይ የኖኑበት ስራ ምንድነው (ገንዘብ ለማግኘት የሚሰሩት ማንኛውም ስራ?) | | | 🞏1= የመንግስት ሰራተኛ  🞏2=የግል/መንግስታዊ ያልሆነ ድርጅት ሰራተኛ 🞏3=የራስ ስራ/እርሻ | | | | | | | 🞏4= የቀን ሰራተኛ  🞏5= የቤትእመቤት  🞏6= ተማሪ  🞏7= ስራ አጥ  🞏8= ሌላ ካለ ይግለጹ _______________ __________ | | | | | | | | |  | |
| 110 | | የእርስዎን ጨምሮ የቤተሰብዎ ገቢ ምን ያህል ይሆናል | | | _________________ ብር/ወር | | | | | | | | | | | | | | | |  | |
| 111 | | ከዚህ በታች ካሉት በእርስዎ ቤት ያለው ምንድነው? (የሀብት መለኪያ) | | | | | | | | | | | | | | | | | | | | |
|  | | መብራት | | | አዎ ------- | | | | | | | | | የለም ------- | | | | | | |  | |
|  |  | የግድግዳ ሰዓት | | | አዎ ------- | | | | | | | | | የለም ------- | | | | | | |  | |
|  |  | ራዲዮ | | | አዎ ------- | | | | | | | | | የለም ------- | | | | | | |  | |
|  |  | ቴሌቪዥን | | | አዎ ------- | | | | | | | | | የለም ------- | | | | | | |  | |
|  | | ሞባይል ስልክ | | | አዎ ------- | | | | | | | | | የለም ------- | | | | | | |  | |
|  |  | ፍሪጅ | | | አዎ ------- | | | | | | | | | የለም ------- | | | | | | |  | |
|  |  | የተለየ ኩሺና ቤት | | | አዎ ------- | | | | | | | | | የለም ------- | | | | | | |  | |
|  |  | ሳይክል | | | አዎ ------- | | | | | | | | | የለም ------- | | | | | | |  | |
|  |  | የባንክ ቁጠባ ሂሳብ | | | አዎ ------- | | | | | | | | | የለም ------- | | | | | | |  | |
|  |  | ሽንት ቤት | | | አዎ ------- | | | | | | | | | የለም ------- | | | | | | |  | |
|  |  | የወለሉ ዓይነት | | | ጭቃ/አፈር------ | | | | | | | | | ሲሚንቶ/እንጨት | | | | | | |  | |
|  |  | የግድግዳው አይነት | | | ግድግዳ የለም /ጭቃ ብቻ _____ | | | | | | | | | ሲሚንቶ/እንጨት/በጭቃ የተለጠፈ _____ | | | | | | |  | |
| 112 | | ዋና ዋና ወጪዎችን (የትምህርት ቤት ክፍያ፣ ምግብ፣ የህክምና ወጪ፣ የቤት ኪራይ… ወዘተ) ለመሸፈን የቤተሰብዎ ዋነኛ የገቢ ምንጭ ምንድነው | | | 🞏1= የራሶት ገቢ 🞏2= የትዳር አጋርዎ ገቢ ወይም የቤተሰብ እርዳታ | | | | | | | | | 🞏3= ከመነሻ ቤተሰብዎ የሚደረግልዎት እርዳታ  🞏4= ሌላ ከሆነ ይግለፁ _______________ | | | | | | |  | |
| 113 | | ከነዚህ አደንዛዥ እፆች በአሁኑ ጊዜ የቱን ይጠቀማሉ | | | 🞏1= ጫት  🞏2= ሲጋራ  🞏3= አልኮሆል | | | | | | | | 🞏4= ሌላ ካለ ይግለፁ _________  🞏5= ምንም አልጠቀምም | | | | | | | |  | |
| **ክፍል 2 የግንኙነት መረጃ፡ የመጨረሻው (የቅርብ ጊዜ)የወሲብ ጓደኛዎት ጋር ያልዎትን ግንኙነት ልጠይቆት ነው፡፡ የመጨረሻውየወሲብ ጓደኛ ማለት በአሁኑ ጊዜ ወይም ለመጨረሻ ጊዜ ከሌሎች ሰዎች ጋር ይልቅ ከርሱ ጋር አብዝተው የግብረስጋ ግንኙነት ይፈፅሙ የነበረው ሰው ማለት ነው፡፡ የሚቆጠቡ ከሆነ ስለ ትዳር አጋርዎ መልስ ይመልሱ፡፡ መደበኛ አጋር ማለት በመደበኛነት የግብረስጋንግኙነት የሚፈጽሙት ሰው ማለት ሆኖ የትዳር አጋር ማለት ግን አይደለም፡፡ መደበኛ ያልሆነ አጋር ማለት አንዴ ወይም ሳይዘወተር የግብረስጋ ግንኙነት የሚፈጽሙት ሰው ማለት ነው፡፡ በአሁኑ ጊዜ በብዛት መደበኛ ያልሆነ ሰው ጋር ያሉ እንደሆነ ስለዚያ ሰው ምላሽ ይስጡ፡፡** | | | | | | | | | | | | | | | | | | | | | | |
| 201 | | በደምዎት ውስጥ ኤች አይ ቪ እንዳለ ባወቁ ቀን ወይም ከዛ ወዲህ የወሲብ ጓደኛ/የትዳር አጋር ነበሮት / ኖርዎት ያውቃል ? | 🞏1= አዎ | | | | | | | 🞏2= የለም | | | | | | | | | ሁሉንም ስለ ወሲብ ጓደኛ የሚጠየቁ ጥያቄዎች ይዝለሉ | | | |
| 202 | | የቅርብ ጊዜ (የመጨረሻው) የወሲብ ጓደኛዎት ጋር የነበሮት ግንኙነት ምን አይነት ነበር?  (√ ያድርጉ) | 🞏1= ያገባ  🞏2= ያላገባ (መደበኛ እጮኛ) | | | | | | | 🞏3= ያላገባ (መደበኛ ያልሆነ የወሲብ አጋር)  🞏4= ያላገባ (የወሲብ አጋር ኖሮኝ አያውቅም) | | | | | | | | | | | ወደ ጥያቄ 301 ይለፉ | |
| 203 | | የመጨረሻው አጋርዎት ጋር ያልዎት/ የነበርዎት ግንኙነት እንዴት ነበር? | 1 = አብረን እየኖርን ነው/እንኖር ነበር | | | | | | | 🞏2 = አብረን አልኖርንም | | | | | | | | | | |  | |
| 204 | | ከአጋርዎ ጋር ለምን ያህል ጊዜ ትተዋወቃላችሁ? | –––––––––– | | | | | | | | | | | | | | | | | |  | |
| 205 | | ግንኙነታችሁ ቆይታው ለምን ያህል ጊዜ እንደሚሆን ይሰማዎት ነበር? | 🞏1= እድሜ ልክ  🞏2= ለረጅም ጊዜ | | | | | | | | | | 🞏3= ለተወሰነ ጊዜእ  🞏4= ለአጭር ጊዜ ብቻ | | | | | | | |  | |
| 206 | | የመጨረሻው አጋርዎ እድሜ ስንት ነው/ ነበር? | ______________ አመት | | | | | | | | | | | | | | | | | |  | |
| 207 | | የመጨረሻው አጋርዎ በማንኛውም ቋንቋ ማንበብና መጻፍ ይችላሉ? | 🞏1= አዎ | | | | | | | | | | 🞏2= የለም | | | | | | | | ወደ ጥያቄ 209 ይለፉ | |
| 208 | | ለላይኛው ጥያቄ መልስዎ አዎ ከሆነ መደበኛ ትምህርት ተከታትለዋል? | 🞏1= አዎ (ደረጃ ______________) | | | | | | | | | | 🞏2= የለም | | | | | | | |  | |
| 209 | | ከመጨረሻ አጋርዎ ጋራ የነበሮትን ግንኙነት እንዴት ይገልፁታል (*ያሳወቁ እንደው ፡ በኤችአይቪ መያዞትን ለ*መጨረሻው የወሲብ አጋርዎ *እስኪያሳዉቁ ብለው ይጠይቁ)* | 🞏1= ሰላማዊ  🞏2= አንዳንዴ እንጋጫለን  🞏3= ብዙ ጊዜ እንጋጫለን | | | | | | | | | | 🞏4= እንጋጫለን ልንለያይ ደርሰን ነበር  🞏5= ሌላ ከሆነ ይግለፁ _________ ______________ | | | | | | | |  | |
| 210 | | ከመመርመርዎ በፊት ከወሲብ ጓደኛዎት ጋር ስለ ኤችአይቪ ምርመራ አውርተው ያውቃሉ ? | 🞏1= አዎ | | | | | | | | | | 🞏2= የለም | | | | | | | | ወደ ጥያቄ 212 ይለፉ | |
| 211 | | በውይይቱ ወቅት አጋርዎት ስለ ኤችአይቪ ምርመራ ማድረግ ላይ የነበራቸው አቋም ምን ይመስል ነበር? | 🞏1= ደስተኛ  🞏2= ግዴለሽ | | | | | | | | | | 🞏3= ተናዳጅ  🞏4= ሌላ ከሆነ ይግለፁ ________ | | | | | | | |  | |
| 212 | | የመጨረሻ አጋርዎት ኤችአይቪ ተመርመረዋል? ውጤቱስ? | 🞏1= ፖዘቲፍ  🞏2= ነጌቲቭ  🞏3= ተመርምረዋል ግን ውጤቱን አላውቀውም | | | | | | | | | | 🞏4= አልተመረመሩም 🞏5= ስለ መመርመራቸው አላውቅም | | | | | | | |  | |
| 213 | | መልስዎ ከላይ ላለው ጥያቄ አላውቅም / አልተመረመሩም ከሆነ ስለ አጋርዎ ኤችአይቪ ሁኔታ ምን ያስባሉ? | 🞏1=ፖዘቲቭ ነው  🞏2= ነጌቲቭ ነው | | | | | | | | | | 🞏3= ይህ ነው ማለት አልችልም | | | | | | | |  | |
| **ክፍል 3፡ ኤች አይ ቪ ኤድስ ምርመራና ሕክምና** | | | | | | | | | | | | | | | | | | | | | | |
| 301 | | ኤችአይቪ ኤድስ ለመመርመር የሄዱበት ዋና ምክንያት ምንድነው? (ከአንድ ምላሽ በላይ መመለስ ይቻላል) | | | 🞏1=አጋሬ ፖዘቲቭ መሆናቸውን ስለነገሩኝ  🞏2= ከዚህ በፊት በነበረኝ የወሲብ ልማድ  🞏3= የአጋሬ የወሲብ ያለፈው ልማድ  🞏4= የትዳር አጋሬ እንድመረመር ስለጠየቁኝ  🞏5=የትዳር አጋሬ ስለታመሙ ወይም ስለሞቱ  🞏6= ስለታማ ነገር ስለወጋኝ ወይም ደም ልገሳ ስለተደረገልኝ | | | | | | | | 🞏7= የኤችአይቪ ኤድስ ህሙማን ስለምንከባከብ  🞏8= ያለሁበትን ሁኔታ ለማወቅ  🞏9= ስላመመኝ  🞏10= ስራ ቦታ/ኤምባሲ ስለተጠየቀ  🞏11= ላገባ ስለነበር  🞏12= ልጅ ለመውለድ እቅድ ስለነበረኝ  🞏13= እርጉዝስለነበርኩኝ ለፅንሱ በመጠንቀቅ  🞏14= ሌላ ከሆነ ይግለፁ | | | | | | | |  | |
| 302 | | ኤች አይ ቪ ለመመርመር ከመወሰንዎ በፊት ለምን ያክል ጊዜ ሲያስቡበት ቆይተዋል | | | 🞏1 = ቀናት  🞏2 = ሳምንታት 🞏3 = ወራት | | | | | | | | 🞏4= አመታት  🞏5= ሌላ ከሆነ ይግለፁ | | | | | | | |  | |
| 303 | | ቫይረሱ በደሞት እንዳለ ተመርመረው ያወቁት መቼ ነው ? | | | _________________(ወር/አመት) | | | | | | | | | | | | | | | |  | |
| 304 | | ከመርመራው በፊት ወይም በኋላ የምክር አገልግሎት አግኝተዋል? | | | 1=🞏አዎ | | | | | | | | 🞏2= የለም | | | | | | | |  | |
| 305 | | ኤችአይቪ ምርመራ ሲያደርጉ ከማን ጋር ነበሩ? | | | 🞏1=ብቻዬን  🞏2= ከትዳር አጋሬ ጋር | | | | | | | | 🞏3= ሌላ ካለ ይግለፁ | | | | | | | |  | |
| 306 | | የምክር አገልግሎት የሰጥዎት ባለሙያ ኤች አይቪ አንዳለቦት ለወሲብ ጓኞች፣ ቤተሰብ አባላትና ሌሎች ጠቃሚ ሰዎች መግለፅ ጥቅም ሊኖረው እንደሚችል አስረድቶዎታል? | | | 🞏1=አዎ | | | | | | | | 🞏2= የለም | | | | | | | |  | |
| 307 | | የምክር አገልግሎት የሰጠዎት ባለሙያ እርሶ ካላሳወቁ ለወሲብ ጓደኛዎት ለማሳወቅ እንደሚገደድ ህጉን ነግርዎታል? | | | 🞏1=አዎ | | | | | | | | 🞏2= የለም | | | | | | | |  | |
| 308 | | የፀረ-ኤችአይቪ መድሃኒት ተጠቃሚ ኖት? | | | 1=🞏አዎ (ረጂመን ___________)  ለምን ያህል ጊዜ ______ ወር/አመት) | | | | | | | | 🞏2= የለም | | | | | | | | ወደ ጥያቄ 312 ይለፉ | |
| 309 | | ባለፈው አንድ ወር ውስጥ የፀረ-ኤችአይቪ መድሃኒትዎን ሳይወስዱ የቀሩበት ምን ያህል ጊዜ ነው? | | | ––––––––– | | | | | | | | | | | | | | | |  | |
| 310 | | ባለፈው አንድ ወር ውስጥ የፀረ-ኤችአይቪ መድሃኒትዎን መውሰድ ባለብዎት ጊዜ በ2 ሰዓት ገደማ ውስጥ ሳይወስዱ የቀሩበት ጊዜ ምን ያህል ነው? | | | ––––––––– | | | | | | | | | | | | | | | |  | |
| 311 | | ባለፉት አንድ ወር ውስጥ ኤች አይቪ በደሞት ውስጥ እንዳለ ከማያውቁ ሰዎች ጋር በመሆኖት እነርሱ እንዳያይዎት ብለው መድሃኒቱን ሳይወስዱ የቀሩበት ጊዜ ወይም የመድሃኒቱን ሰዓት ያሳለፉበት ጊዜ ምን ያህል ነው ? | | | 🞏1= በፍጹም  🞏2= አንዳንድ ጊዜ | | | | | | | | 🞏3= ብዙ ጊዜ | | | | | | | |  | |
| 312 | | ቢያንስ የአንድ የኤችአይቪ ህመምተኞች ማህበር አባል ኖት? | | | 🞏1=አዎ | | | | | | | | 🞏2= የለም | | | | | | | |  | |
| 313 | | የህመሙ ደረጃ (የታካሚውን ካርድ ይመልከቱ) | | | 🞏1=ደረጃ 1  🞏2= ደረጃ 2  🞏3= ደረጃ3 | | | | | | | | 🞏4= ደረጃ 4  🞏5= አልተገለፀም | | | | | | | |  | |
| **ክፍል 4፡ በኤችአይቪ መያዝ ሁኔታ ማሳወቅና ደንቃራዎቹ (ሁለቱንም ሳጥን መሙላት አይርሱ)** | | | | | | | | | | | | | | | | | | | | | | |
| 401 | የቅርብ ሰው (ምርመራውን ካደረገልዎት ጤና ባለሞያ ውጪ) በኤችአይቪ መያዞትን የሚያውቅ አለ? | | | 🞏1=አዎ | | | | | | | | | 🞏2= የለም | | | | | | | | ወደ ጥያቄ 411 ይለፉ | |
| 402 | ከላይ ለተነሳው ጥያቄ መልስዎ አዎ ከሆነ ከኤችአይቪ ጋር እንደሚኖሩ የሚያውቅ ማነው?  (ከአንድ በላይ መልስ ማስቀመጥ ይቻላል) | | | 🞏1= የቅርብ ጊዜ የወሲብ አጋሬ  🞏2 = እናቴ  🞏3= አባቴ  🞏4= ልጄ  🞏5 = እህት/ወንድሜ  🞏6 = ሌሎች የቤተሰቤ አባላት | | | | | | | | | 🞏7 = ዘመዴ  🞏8 = ጓደኛዬ  🞏9 = የሃይማኖት መሪ  🞏10 = ጎረቤት  🞏11= ብዙ ጊዜ የሚያክሙኝ የህክምና ባለሙያዎች  🞏12 = ሌላ ካለ የግለፁ | | | | | | | |  | |
| 403 | የመጨረሻው የወሲብ አጋርዎ **(የመጀመሪያውን ሳጥን** ምልክ ትያድርጉበት) እና ከቤተሰብዎት/ከቅርብ ዘመድዎች ውስጥ መጀመሪያ ያወቀው ሰው **(ሁለተኛውን ሳጥን** ምልክት ያድርጉበት) እርስዎ የኤችአየቪ ፖዘቲቭ መሆንዎን እንዴት ሊያውቁ ቻሉ? | | | **🞏🞏**1=በራሴ ስለነገርኳቸው  **🞏🞏**2= ከሌላ ሰው ጋር ሆኜ ስለነገርኳቸው  **🞏🞏**3= ሌላ ሰው እንዲነግራቸው ስላደረኩኝ | | | | | | | | | **🞏🞏**4= የትዳር አጋሬ የተሰጠኝን የህክምና መድሃኒቶች/የበሽታው ምልክት/የምርመራ ወረቀት… ወዘተ አይተው ስለጠየቁኝ ነገርኳቸው  **🞏🞏**5= ከ3ኛ ወገን | | | | | | | |  | |
| 404 | ኤችአይቪ ኤድስ ምርመራ ካደረጉ በኋላ ወይም የወሲብ አጋርዎ ጋር ግንኙነት ከጀመሩ በኋላ (ጓደንነት ከመጀመርዎ በፊት ተመርምረው ፖዘቲቭ የሆኑ እንደሆነ) ለምን ያህል ጊዜ ቆይተው ለወሲብ አጋርዎ ነገሯቸው? | | | በተቻለ መጠን ቀኑን፣ ሳምንቱን፣ ወሩን እና አመቱን ይግለፁ | | | | | | | | | | | | | | | | |  | |
| 405 | የኤችአይቪ ኤድስ ምርመራ ካደረጉ በኋላ ለመጀመሪያ ጊዜ ለቤተሰብዎ ወይም ለቅርብ ዘመድዎ ለምን ያክል ጊዜ ቆይተው አሳወቁ | | | በተቻለ መጠን ቀኑን፣ ሳምንቱን፣ ወሩን እና አመቱን ይግለፁ | | | | | | | | | | | | | | | | |  | |
| 406 | ኤችአይቪ በደምዎ ውስጥ እንዳለ ካወቁ በኋላ እንዳለብዎት ሳያሳውቁ ከትዳር አጋርዎ ጋር የግብረስጋ ግንኙነት ፈፅመው ያውቃሉ? | | | 🞏1=አዎ | | | | | | | | | 🞏2= የለም | | | | | | | |  | |
| 407 | ለመጨረሻው የወሲብ ጓደኛዎት **(የመጀሪያውን ሳጥን** ምልክት ያድርጉበት) እና ከቤተሰብ ወይም የቅርብ ዘመድ ለነገሩት ለመጀመሪያው ሰው **(የሁለተኛውን ሳጥን** ምልክት ያድርጉበት) የኤችአይቪ ቫይረስ በደምዎ ውስጥ እንዳለ የነገሩበት ምክንያት ምንድነው? (ከአንድ በላይ መልስ ማስቀመጥ ይቻላል) | | | **🞏🞏**1= የምክር አገልግሎት ሰጪው አበረታቶኝ ነው  **🞏🞏**2= አደጋ ላይ እንዳይወድቁ ስለማልፈልግ ነው **🞏🞏3**= ድጋፋቸውን ስለምፈልግ  **🞏🞏**4= ማንኛውም ምስጢራዊ ጉዳዮቼን ስለማዋያቸው | | | | | | | | | **🞏🞏**5= በህግ መከሰስ ስለማልፈልፈልግ  **🞏🞏**6= እንዲህ ያለ ነገር ለመደበቅ እግዚአብሔርን ፈርቼ ነው  **🞏🞏**7= ሌላ ካለ ይግለፁ ________ _______________ _______________ | | | | | | | |  | |
| 408 | የመጨረሻው የወሲብ አጋርዎ **(የመጀመሪያውን ሳጥን** ምልክት ያድርጉበት) እና መጀመሪያ ያወቀው ቤተሰብ/የቅርብ ዘመድዎ **(የሁለተኛውን ሳጥን** ምልክት ያድርጉበት) እርስዎ በደምዎ ውስጥ ቫይረሱ እንዳለብዎ ሲያውቁ የነበራቸው ምላሽ ምን ይመስል ነበር? (ከአንድ በላይ መልስ ማስቀመጥ ይቻላል) | | | **🞏🞏**1= ድጋፍ አድርገውልኛል (ሀሳባዊ ወይም የገንዘብ)  **🞏🞏**2=ስሜታቸው ተጎድቷል (ግራ መጋባት/ሀዘን/ንዴት**🞏🞏**3= የፀረ-ኤችአይቪ ህክምናና ክትትል ለማድረግ ነፃነት ሰጡኝ  **🞏🞏**4= አካላዊ ጥቃት  **🞏🞏**5= የቃላት ጥቃትና ዛቻ | | | | | | | | | **🞏🞏**6= መገለልና መድልኦ አደረሱብኝ  **🞏🞏**7= ስለራሳቸው የኤችአይቪ ሁኔታ ተጨነቁ  **🞏🞏**8= ያለፈ የወሲብ ታሪኬን ጠየቁኝ  **🞏🞏**9=ግንኙነታችንን አቋረጡት  **🞏🞏**10= ሌላ ካለ ይግለፁ _________ _______________ | | | | | | | |  | |
| 409 | የመጨረሻው የወሲብ አጋርዎት **(የመጀመሪያውን ሳጥን** ምልክት ያድርጉበት) ከቅርብ ዘመድ ወይም ቤተሰብ መጀመሪያ የነገሩት ሰው እርሶ ቫይረሱ በደምዎ ውስጥ እንዳለ ልክ እንዳወቁ **(የሁለተኛውን ሳጥን** ምልክት ያድርጉበት) ቫይረሱ በደምዎ ውስጥ እንዳለ ልክ እንዳወቁ ያላቸው ምላሽ እንዴት ይነፃፀራል? | | | **🞏🞏**1=መጀመሪያም ምላሻቸው የድጋፍ ነበር - አሁንም ምላሻቸው የድጋፍ ነው  **🞏🞏**2= መጀመሪያ የድጋፍ ነበር አሁን ምላሻቸው የድጋፍ አልሆነም/ጎድቶኛል | | | | | | | | | **🞏🞏**3= መጀመሪያ ምላሻቸው የድጋፍ አልነበረም/ጎጂ ነበር አሁን ግን ጠቃሚ ነው  **🞏🞏**4= መጀመሪያም ምላሻቸው የድጋፍ አልነበረም/ጎድቶኛል አሁንም ምላሻቸው የድጋፍ ይደለም/ጎጂ ነው | | | | | | | |  | |
| 410 | እርስዎ ኤችአይቪ ቫይረስ በደምዎ ውስጥ እንዳለብዎት ካሳወቁ በኋላ እርስዎም ሆነ አሁን ያልዎት የወሲብ አጋርዎ ጥንቃቄ የተሞላበት ግብረስጋ ግንኙነት ውይይት አደረጋችሁ? | | | 🞏1= አዎ | | | | | | | | | 🞏2= የለም | | | | | | | |  | |
| 411 | ለመጨረሻው የወሲብ አጋርዎ **(የመጀመሪያውን ሳጥን** ምልክት ያድርጉበት) እና ለቤተሰብ/ቅርብ ዘመድ**(የሁለተኛውን ሳጥን** ምልክት ያድርጉበት) እርስዎ በደምዎ ውስጥ ኤችአይቪ ቫይረስ እንዳለ ያላሳወቁበት ምክንያት ምንምድነው? (ከአንድ በላይ መልስ ማስቀመጥ ይቻላል) | | | **🞏🞏**1=ግንኙነታችን ሊቋረጥ ይችላል በሚል  **🞏🞏**2= ግለሰቡ ከእኔ በሽታው እንዳይዘው ይፈራል ብዬ  **🞏🞏**3= ግለሰቡ በቃላት ይጎዳኛል ወይም ያጠቃኛል ብዬ ስለምፈራ  **🞏🞏**4 = ግለሰቡ መረጃውን ለመስማት እድሜው ገና ስለሆነ  **🞏🞏**5= ግለሰቡ ለ3ኛ ወገን ሊናገር ስለሚችል  **🞏🞏**6= ግለሰቡ በአሁኑ ሰዓት በርካታ የግል ችግሮች ስላሉበት | | | | | | | | | **🞏🞏**7= መናገሩ አስፈላጊ ስላልሆነ  **🞏🞏**8= ግለሰቡን ማስጨነቅ ስለማልፈልግነ  **🞏🞏**9= ግለሰቡ የአካል ጥቃት እንዳያደርስብኝ  **🞏🞏**10= ግለሰቡ ሊገለኝ ስለሚችል  **🞏🞏**11= ግለሰቡ እኔን አመንዝረኛ አድርጎ እንዳይቆጥረኝ  **🞏🞏**12= ሌላ ካለ ይግለፁ | | | | | | | |  | |
| **ክፍል 5፡ የግብረ ስጋ ስነ- ባህሪይ እና ልማድ** | | | | | | | | | | | | | | | | | | | | | | |
| 501 | በህይወት ዘመንዎ ስንት የወሲብ ጓደኞች ኖሮት ያውቃል ? | | | _____________________________ | | | | | | | | | | | | | | | | |  | |
| 502 | በአሁኑ ጊዜ ስንት የወሲብ ጓደኞች አልዎት? | | | _____________________________ | | | | | | | | | | | | | | | | |  | |
| 503 | ከመጨረሻው የወሲብ አጋርዎት ጋር እርስዎ ኤችአይቪ ኤድስ በደምዎ ውስጥ እንዳለ ካወቁ በኋላ የግብስጋ ግንኙነት ሲፈፅሙ ኮንዶም ለምን ያህል ጊዜ ይጠቀማሉ? | | | 🞏1= ሁል ጊዜ (100%)  🞏2= ብዙ ጊዜ 🞏3= አንድ አንድ ጊዜ | | | | | | | | | 🞏4= በጭራሽ አልጠቀምም  🞏5= እቆጠባለሁ | | | | | | | |  | |
| 504 | ከመጨረሻው የወሲብ አጋርዎት ጋር ኮንዶም 100% የማይጠቀሙ ከሆነ ለዚህ ምክንያትዎ ምንድነው? (ከአንድ በላይ መልስ ማስቀመጥ ይቻላል) | | | 🞏1= ባልደረባዬ ኤችአይቪ ኤድስ በደሙ ውስጥ ስለሚገኝ  🞏2= ኮንዶም መጠቀም ደስታን ስለሚቀንስ  🞏3= ኮንዶም ብጠቀም ባልደረባዬ ኤችአይቪ በደሜ ውስጥ እንዳለ ስለሚጠረጥሩ | | | | | | | | | 🞏4= አጠቃቀሙን ስለማልችልበት  🞏5= ስለ ኮንዶም ውይይት ማድረግ ከባድ ሆኖ ስላገኘሁት  🞏6= የኮንዶም አቅርቦት ስለሌለኝ 🞏7= ሌላ ካለ ይግለፁ | | | | | | | |  | |
| 505 | ከመጨረሻው የወሲብ አጋርዎት ጋር ምን አይነት የወሊድ መቆጣጠሪያ ዘዴዎች ይጠቀማሉ? (ከአንድ በላይ መልስ ማስቀመጥ ይቻላል) | | | 🞏1= ምንም አይነት መቆጣጠሪያ አንጠቀምም  🞏2= ፒልስ  🞏3= መርፌውን 🞏4= ኖርፕላንት | | | | | | | | | 🞏5= ዲያፍራም  🞏6= ኮንዶም  🞏7= እንዳይወለድ ተደርጓል (አስቴሪያላይዜሽን)  🞏8= ሌላ ካለ ይግለፁ | | | | | | | |  | |
| 506 | ምንም የወሊድ መቆጣጠሪያ ዘዴ የማይጠቀሙ ከሆነ የማይጠቀሙበት ምክንያትዎ ምንድነው? | | | 🞏1= ስለምንታቀብ 🞏2= ልጅ መውለድ ስለምንፈልግ | | | | | | | | | 🞏3= ሌላ ካለ ይግለፁ ________ _______________ _______________ | | | | | | | |  | |
| መደበኛ ያልሆነ አጋር (ይህ ጥያቄ የሚመለከተው የመጨረሻው አጋር የትዳር ባልደረባ ወይም መደበኛ ባልደረባ ለሆነው ሰው ነው) አሁን መደበኛ ስላልሆነው የትዳር አጋርዎ ጥያቄ አነሳልዎታለሁ፡፡ የግብረስጋ ግንኙነት ባልደረባዎ ያላገቡትና አብረው ኖረው የማያውቁት፡፡ መደበኛ ያልሆነ አጋር ማለት የግብረስጋ ግንኙነት አንድ ጊዜ ብቻ ወይም በጣም አልፎ አልፎ የሚፈፅሙት ሰው ማለት ነው፡፡ | | | | | | | | | | | | | | | | | | | | | | |
| 507 | ባለፈው አንድ አመት ውስጥ መደበኛ ጓደኛ/ባለቤትዎ ካልሆነ ሰው ጋር ግብረስጋ ግንኙነት ፈፅመው ያውቃሉ? | | | 🞏1= አዎ | | | | | | | | | 🞏2= የለም | | | | | | | | ወደ ጥያቄ 601 ይለፉ | |
| 508 | ለመጨረሻ ጊዜ የነበርዎት መደበኛ ያልሆነ የወሲብ አጋር የኤች አይ ቪ ሁኔታቸው ምን ይመስላል? | | | 🞏1= ፖዘቲቭ  🞏2= ነጌቲቭ | | | | | | | | | 🞏3= አላውቅም | | | | | | | |  | |
| 509 | ከላይ ለተነሳው ጥያቄ መልስዎ አላውቅም ከሆነ የመጨረሻው መደበኛ ያልሆነ የወሲብ አጋርዎ የኤችአይቪ ሁኔታ ምን ይሆናል ብለው ያስባሉ? | | | 🞏1= ፖዘቲቭ  🞏2= ነጌቲቭ | | | | | | | | | 🞏3= ይህ ነው ማለት አልችልም | | | | | | | |  | |
| 510 | ለመጨረሻ ጊዜ የነበርዎት መደበኛ ያልሆነ የወሲብ አጋርዎ ስለእርስዎ ኤችአይቪ በደምዎ ውስጥ መኖር ያውቃሉ? | | | 🞏1= አዎ | | | | | | | | | 🞏2= የለም | | | | | | | | ወደ ጥያቄ 512ይለፉ | |
| 511 | ከላይ ለተነሳው ጥያቄ መልስዎ አዎ ከሆነ እንዴት ሊያውቁ ቻሉ? | | | 🞏1= እኔ አሳውቄው  🞏2= ከ3ኛ ወገን ሰምተው | | | | | | | | | 🞏3= የህክምና ሪያሊቲዎቼን/የበሽታውን ምልክት/ የመርመራውን ውጤት… ወዘተ አይተው ሲጠይቁኝ ነገርኳቸው | | | | | | | |  | |
| 512 | አሁን ካልዎት መደበኛ ያልሆነ የግብረስጋ ባልደረባዎ ጋር ኮንዶም ምን ያህል ጊዜ ይጠቀማሉ? | | | 🞏1= ሁል ጊዜ (100%)  🞏2= ብዙ ጊዜ | | | | | | | | | 🞏3= አንዳንድ ጊዜ 🞏4= በፍጹም ተጠቅመን አናውቅም | | | | | | | |  | |
| **ክፍል 6፡ ማህበራዊ ድጋፍ (የኦስሎ ማህበራዊ ድጋፍ ልኬት)** | | | | | | | | | | | | | | | | | | | | | | |
| 601 | ችግር ሲደርስብዎ የሚደርስልዎት የቅርብ ሰው ምን ያህል አልዎት? | | | | | | የለም | | | | | | | | | | | | | | 1 |  |
|  |  |  |  |  |  |  | 1 ወይም 2 | | | | | | | | | | | | | | 2 |  |
|  |  |  |  |  |  |  | 3-5 | | | | | | | | | | | | | | 3 |  |
|  |  |  |  |  |  |  | 6 ወይም ከዚያ በላይ | | | | | | | | | | | | | | 4 |  |
| 602 | እርስዎ በሚያደርጉት ነገር ሰዎች ምን ያክል ትኩረት ወይም ፍላጎት ይኖራቸዋል? | | | | | | ብዙ ትኩረትና ፍላጎት | | | | | | | | | | | | | | 5 |  |
|  |  |  |  |  |  |  | አንዳንድ ትኩረትና ፍላጎት | | | | | | | | | | | | | | 4 |  |
|  |  |  |  |  |  |  | እርግጠኛ አይደለም | | | | | | | | | | | | | | 3 |  |
|  |  |  |  |  |  |  | ውስን ትኩረትና ፍላጎት | | | | | | | | | | | | | | 2 |  |
|  |  |  |  |  |  |  | ምንም አይነት ትኩረትና ፍላጎት የለም | | | | | | | | | | | | | | 1 |  |
| 603 | እርዳታ በሚያስፈልግዎት ጊዜ ከጎረቤትዎ እርዳታ የሚያገኙበት ሁኔታ ምን ይመስላል? | | | | | | በጣም ቀላል | | | | | | | | | | | | | | 5 |  |
|  |  |  |  |  |  |  | ቀላል | | | | | | | | | | | | | | 4 |  |
|  |  |  |  |  |  |  | ይቻላል | | | | | | | | | | | | | | 3 |  |
|  |  |  |  |  |  |  | ከባድ | | | | | | | | | | | | | | 2 |  |
|  |  |  |  |  |  |  | በጣም ከባድ | | | | | | | | | | | | | | 1 |  |
| **ክፍል 7 የድብርት ልኬት፡ (የታካሚው ጤና መጠይቅ 9) ባለፉት ሁለት ሳምንታት ውስጥ ከዚህ በታች ካሉት ችግሮች ውስጥ በየትኛው ተጨንቀዋል?** | | | | | | | | | | | | | | | | | | | | | | |
|  |  | | | | | በጭራሽ | | | አንዳንድ ቀናት | | | | | ብዙቀናት | | | | ከሞላጎደል በየቀኑ | | |  | |
| 702 | ነገሮችን በማከናወን ወቅት ደስታ ወይም ፍላጎት ማጣት | | | | | 0 | | | 1 | | | | | 2 | | | | 3 | | |  | |
| 703 | ድብርት ወይም ተስፋ የመቁረጥ ስሜት | | | | | 0 | | | 1 | | | | | 2 | | | | 3 | | |  | |
| 704 | መተኛት ወይም ማረፍ አለመቻል ወይም ከመጠን በላይ መተኛት | | | | | 0 | | | 1 | | | | | 2 | | | | 3 | | |  | |
| 705 | በጣም መድከም ወይም ውስን አቅም መኖር | | | | | 0 | | | 1 | | | | | 2 | | | | 3 | | |  | |
| 706 | የምግብ ፍላጎት ማነስ ወይም በጣም መጨመር | | | | | 0 | | | 1 | | | | | 2 | | | | 3 | | |  | |
| 707 | ስለራስ መጥፎ ስሜት መሰማት ወይም እራስዎን ወይም ቤተሰብዎን ለውድቅት የዳረጉ መስሎ መሰማት | | | | | 0 | | | 1 | | | | | 2 | | | | 3 | | |  | |
| 708 | ትኩረት ማነስ ለምሳሌ ጋዜጣ ማንበብ እና ቴሌቪዥን ማየት ላይ ትኩረት ማነስ | | | | | 0 | | | 1 | | | | | 2 | | | | 3 | | |  | |
| 709 | ሌሎች ሰዎች እስኪያስተውሉት ድረስ እጅግ በጣም ዝግ ብሎ መንቀሳቀስ ወይም መናገር ወይም በተቃራኒው መቁነጥነጥ ወይም እረፍት ማጣት ከተለመደው በላይ እንቅስቃሴ በማድረግ | | | | | 0 | | | 1 | | | | | 2 | | | | 3 | | |  | |
| 710 | ቢሞቱ ወይም እራስዎን ቢጎዱ መምረጥ | | | | | 0 | | | 1 | | | | | 2 | | | | 3 | | |  | |
|  | ወደታች መስመር ይደምሩ | | | | |  | | |  | | | | |  | | | |  | | |  | |
|  |  | | | | | ድምር | | |  | | | | | | | | | | | |  | |
| 711 | ከላይ ከተጠቀሱት ችግሮች ውስጥ ቢያንስ አንዱ ያስቸግረኛል ብለው እንደሆነ ስራ እንዳይሰሩ፣ እቤት ያሉትን ጉዳዮች እንዳያከናውኑ፣ ከሌላ ሰው ጋራ እንዳይሆኑ ምን ያክል ጊዜ አስቸግሮታል ? (ምልክት ያድርጉ) | | | | | 🞏 በጭራሽ ከባድ አይደለም  🞏 የተወሰነ ይከብዳል | | | | | | | | | 🞏 በጣም ከባድ ነው  🞏 እጅግ በጣም ከባድ ነው | | | | | |  | |
| **ክፍል 8 ምናባዊ መድልኦና መገለል፡** | | | | | | | | | | | | | | | | | | | | | | |
| እዚህ ክፍል ውስጥ የተካተቱ ጥያቄዎች ኤችአይቪ ኤድስ በደማቸው ውስጥ ያለባቸው ሰዎች ያላቸውን ስሜትና የሚደረግላቸውን እንክብካቤ አስመልክቶ እርስዎ ያልዎትን ስሜትና አስተያየት ለመጠየቅ የተዘጋጁ ናቸው፡፡ ትክክለኛ የሆነ ወይም ያልሆነ መልስ የለም፡፡ የሚያስቡትን ሁሉ ለመናገር ምንም አይስጉ ከዚህ በታች ያሉትን ጥያቄዎች አስመልክቶ እስማማለሁ ወይም አልስማማም በማለት መልስዎን ያስቀምጡ፡፡ | | | | | | | | | | | | | | | | | | | | | | |
|  |  | | | | | | | በጣም አልስማማም | | | አልስማማም | | | | | | እስማማለሁ | | | በጣም እስማማለሁ | | |
| 801 | በብዙ የህይወት ማዘውተሪያዬ ቦታ አካባቢ ማንም ኤችአይቪ በደሜ ውስጥ መኖሩን አያውቅም | | | | | | | 1=🞏 | | | 2=🞏 | | | | | | 3=🞏 | | | 4=🞏 | | |
| 802 | ኤችአይቪ ኤድስ በደሜ ውስጥ ስላለ የጥፋተኝነት ስሜት ይሰማኛል | | | | | | | 1=🞏 | | | 2=🞏 | | | | | | 3=🞏 | | | 4=🞏 | | |
| 803 | ሰዎች በኤችአይቪ ላይ ያላቸው አመለካከት ስለራሴ መጥፎነት እንዳስብ አድርጎኛል | | | | | | | 1=🞏 | | | 2=🞏 | | | | | | 3=🞏 | | | 4=🞏 | | |
| 804 | ኤችአይቪ በደሜ ውስጥ እንዳለብኝ ለሰዎች መናገር አደጋ አለው | | | | | | | 1=🞏 | | | 2=🞏 | | | | | | 3=🞏 | | | 4=🞏 | | |
| 805 | ኤችአይቪ በደማቸው ውስጥ ያለባቸው ሰዎች አሰሪዎቻቸው ካወቁባቸው ከስራ ይባረራሉ | | | | | | | 1=🞏 | | | 2=🞏 | | | | | | 3=🞏 | | | 4=🞏 | | |
| 806 | ኤችአይቪ በደሜ ውስጥ እንዳለበኝ ለመደበቅ በጣም እጠ ነቀቃለሁ | | | | | | | 1=🞏 | | | 2=🞏 | | | | | | 3=🞏 | | | 4=🞏 | | |
| 807 | ኤችአይቪ ኤድስ በደሜ ውስጥ ስላለብኝ ከሌሎች ሰዎች በታች የሆንኩ ይመስለኛል | | | | | | | 1=🞏 | | | 2=🞏 | | | | | | 3=🞏 | | | 4=🞏 | | |
| 808 | ኤችአይቪ ኤድስ በደሜ ውስጥ ስላለብኝ በፍጹም ላፍርበትም | | | | | | | 4=🞏 | | | 3=🞏 | | | | | | 2=🞏 | | | 1=🞏 | | |
| 809 | ኤችአይቪ በደማቸው ውስጥ ያለባቸው ሰዎች ይገደላሉ | | | | | | | 1=🞏 | | | 2=🞏 | | | | | | 3=🞏 | | | 4=🞏 | | |
| 810 | ኤችአይቪ በደማቸው ውስጥ ያለባቸውን ሰዎች ብዙ ሰዎች እንደቆሻሻ ይቆጥሯቸዋል | | | | | | | 1=🞏 | | | 2=🞏 | | | | | | 3=🞏 | | | 4=🞏 | | |
| 811 | ኤች አይ ቪ በደሜ ውስጥ እንዳለብኝ ከማሳወቅ ይልቅ ያለኝን ግንኙነት ማቋረጥ ይቀለኛል | | | | | | | 1=🞏 | | | 2=🞏 | | | | | | 3=🞏 | | | 4=🞏 | | |
| 812 | ኤችአይቪ በደሜ ውስጥ መኖሩ ንፁህ እንዳልሆንኩ እንዲሰማኝ ያደርገኛል | | | | | | | 1=🞏 | | | 2=🞏 | | | | | | 3=🞏 | | | 4=🞏 | | |
| 813 | ኤችአይቪ በደሜ ውስጥ እንዳለብኝ ካወኩኝ ዘንዳ ከተቀረው አለም የተገነጠልኩና ብቻዬን ያለሁ መስሎ ይሰማኛል | | | | | | | 1=🞏 | | | 2=🞏 | | | | | | 3=🞏 | | | 4=🞏 | | |
| 814 | ብዙ ሰዎች ኤችአይቪ በደሙ ውስጥ ያለበት ሰው አስጠሊታ ነው ብለው ያስባሉ | | | | | | | 1=🞏 | | | 2=🞏 | | | | | | 3=🞏 | | | 4=🞏 | | |
| 815 | ኤችአይቪ በደሜ ውስጥ መኖሩ መጥፎ ሰው እንደሆንኩ እንዲሰማኝ ያደርገኛል | | | | | | | 1=🞏 | | | 2=🞏 | | | | | | 3=🞏 | | | 4=🞏 | | |
| 816 | ኤችአይቪ በደማቸው ውስጥ ያለባቸው ሰዎች ሲታወቅባቸው ይጠላሉ | | | | | | | 1=🞏 | | | 2=🞏 | | | | | | 3=🞏 | | | 4=🞏 | | |
| 817 | ኤችአይቪ በደሜ ውስጥ እንዳለብኝ ለማን መናገር እንዳለብኝ በጣም እጠነቀቃለሁ | | | | | | | 1=🞏 | | | 2=🞏 | | | | | | 3=🞏 | | | 4=🞏 | | |
| 818 | ኤችአይቪ በደሜ ውስጥ እንዳለብኝ ያወቁ ሰዎች ይርቁኛል | | | | | | | 1=🞏 | | | 2=🞏 | | | | | | 3=🞏 | | | 4=🞏 | | |
| 819 | ኤችአይቪ በደሜ ውስጥ እንዳለብኝ ካወኩኝ ዘንዳ ስለሚያገሉኝ ሰዎች እጨነቃለሁ | | | | | | | 1=🞏 | | | 2=🞏 | | | | | | 3=🞏 | | | 4=🞏 | | |
| 820 | ብዙ ሰዎች ከእንዲህ አይነት ሰዎች ጋር ሲሆኑ ምቾት አይሰማቸውም | | | | | | | 1=🞏 | | | 2=🞏 | | | | | | 3=🞏 | | | 4=🞏 | | |
| 821 | ኤችአይቪ በደሜ ውስጥ እንዳለብኝ መደበቅ እንደሚያስፈልግ አስቤው አላውቅም | | | | | | | 4=🞏 | | | 3=🞏 | | | | | | 2=🞏 | | | 1=🞏 | | |
| 822 | ኤችአይቪ ኤድስ እንዳለብኝ ሰዎች ካወቁብኝ ይፈርዱብኛል ብዬ እጨነቃለሁ | | | | | | | 1=🞏 | | | 2=🞏 | | | | | | 3=🞏 | | | 4=🞏 | | |
| 823 | በደሜ ውስጥ ኤችአይቪ ኤድስ መኖሩ ያስጠላኛል | | | | | | | 1=🞏 | | | 2=🞏 | | | | | | 3=🞏 | | | 4=🞏 | | |
| **ክፍል 9 ምናባዊ ደንቃራዎችና ኤችአይቪ ኤድስ ሁኔታን ለማሳወቅ ያለው የግል ብቃት** | | | | | | | | | | | | | | | | | | | | | | |
|  |  | | | | | | | በጣም አልስማማም | | | አልስማማማም | | | | | | እስማማለሁ | | | በጣም እስማማለሁ | | |
| 901 | ኤችአይቪ በደሜ ውስጥ እንዳለብኝ ካሳወኩኝ በገለልና መድልኦ ይደርስብኛል | | | | | | | 1=🞏 | | | 2=🞏 | | | | | | 3=🞏 | | | 4=🞏 | | |
| 902 | የኤችአይቪ ኤድስ ምርመራ ውጤቴን ካሳወኩኝ የትዳር አጋሬ/ቤተሰቦቼ ሊተውኝ ይችላሉ | | | | | | | 1=🞏 | | | 2=🞏 | | | | | | 3=🞏 | | | 4=🞏 | | |
| 903 | ምንም አይነት መገለል ቢደርስብኝም የኤችአይቪ ኤድስ ሁኔታዬን ለማሳወቅ እንደምች እርግጠኛ ነኝ | | | | | | | 1=🞏 | | | 2=🞏 | | | | | | 3=🞏 | | | 4=🞏 | | |
| 904 | ምንም እንኳን የትዳር አጋሬ/ቤተሰቦቼ ቢተውኝም የኤችአይቪ ሁኔታዬን ማሳወቅ እንደምችል እርግጠኛ ነኝ | | | | | | | 1=🞏 | | | 2=🞏 | | | | | | 3=🞏 | | | 4=🞏 | | |
| **ክፍል 10 የወደፊቱ እቅድ** | | | | | | | | | | | | | | | | | | | | | | |
| 1001 | ኤችአይቪ በደምዎ ውስጥ እንዳለ ለወሲብ ጓደኛዎ/ባለቤትዎ ፣ ቤተሰብ ወይም ለሌላ ቅርብ ሰው ለማሳወቅ እቅድ አልዎት? | | | | | | | 🞏1= አዎ | | | | | | | | 🞏2= የለም | | | |  | | |
| **ክፍል 11 በእርግዝና ወቅት የሚደረግ የፀረ-ኤችአይቪ ህክምና (ከአሁን በኋላ የምጠይቅዎት ከእርስዎ ወይም በትዳር አጋርዎት ደም ውስጥ ኤች አይ ቪ መኖሩ በምርመራ ከተረጋገጠ በኋላ ስለተከሰቱ እርግዝናዎች ብቻ ነው)** | | | | | | | | | | | | | | | | | | | | | | |
| 1101 | ከሁለት አንዳችሁ ኤችአይቪ በደማችሁ ውስጥ እንዳለ ካወቃችሁ በኋላ በመካከላችሁ እርግዝና ተከስቶ ያውቃል ? | | | | | | | 🞏1= አዎ (ስንት __________) | | | | | | | | 🞏2= የለም | | | | እዚሁ ይጨርሱ | | |
| 1102 | ከላይ ለተነሳው ጥያቄ መልስዎ አዎ ከሆነ የትዳር አጋርዎ እርስዎ ኤችአይቪ በደምዎ ውስጥ እንዳለብዎት ያወቁት ከእርግዝናው በፊት ነው? ወይስ በእርግዝናው ወቅት ነው? | | | | | | | 🞏1=ከእርግዝናው በፊት | | | | | | | | 🞏2= በእርግዝና ወቅት | | | |  | | |
| 1103 | ሳይታቀድ የተከሰተ እርግዝና ነበር? | | | | | | | 🞏1= አዎ | | | | | | | | 🞏2= የለም | | | |  | | |
| 1104 | አንቺ/ነፍሰጡር አጋርህ በእርግዝና /በወሊድ ወቀት ከእናት ወደ ልጅ ቫይረሱ እንዳይተላለፍ የህክምና ክትትል አድርገሻል/አድርገሃል? | | | | | | | 🞏1= አዎ  🞏2= የለም | | | | | | | | 🞏3= ዉርጃ ነበር  🞏4= ሌላ ከሆነ ይግለፁ | | | |  | | |
| 1105 | በእርግዝና/በወሊድ ወቀት ከእናት ወደ ልጅ ቫይረሱ እንዳይተላለፍ ፀረኤችአይቪ መድኃኒት ልጁ ወስድዋል? | | | | | | | 🞏1= አዎ  🞏2= የለም | | | | | | | | 🞏3= ዉርጃ ነበር  🞏4= ሌላ ከሆነ ይግለፁ | | | |  | | |
| 1106 | አንቺ/ነፍሰጡር አጋርህ ጡት አጠቡ? | | | | | | | 🞏1= አዎ | | | | | | | | 🞏2= የለም | | | |  | | |

መጨረሻ - አመሰግናለሁ !
